# MR Image Changes of Normal-Appearing Brain Tissue after Radiotherapy

**DOI:** 10.3390/cancers13071573

**Published:** 2021-03-29

**Authors:** Katharina Witzmann, Felix Raschke, Esther G. C. Troost

**Affiliations:** 1 Helmholtz-Zentrum Dresden-Rossendorf, Institute of Radiooncology—OncoRay, Dresden, Germany; Katharina.Witzmann@uniklinikum-dresden.de (K.W.); felix.raschke@oncoray.de (F.R.); 2 OncoRay—National Center for Radiation Research in Oncology, Faculty of Medicine and University Hospital Carl Gustav Carus, Technische Universität Dresden, Helmholtz-Zentrum Dresden-Rossendorf, Dresden, Germany; 3 Department of Radiotherapy and Radiation Oncology, Faculty of Medicine and University Hospital Carl Gustav Carus, Technische Universität Dresden, Dresden, Germany; 4 German Cancer Consortium (DKTK), Partner Site Dresden, and German Cancer Research Center (DKFZ), Heidelberg, Germany; 5 National Center for Tumor Diseases (NCT), Partner Site Dresden of the German Cancer Research Center (DKFZ), Faculty of Medicine and University Hospital Carl Gustav Carus, Technische Universität Dresden and Helmholtz Association/Helmholtz-Zentrum Dresden-Rossendorf (HZDR), Dresden, Germany

**Keywords:** radiotherapy, radiation-induced brain injuries, normal-appearing brain tissue, functional MRI, anatomical MRI, perfusion, diffusion, spectroscopy, atrophy

## Abstract

**Simple Summary:**

Radiotherapy is one of the most important treatment options against cancer. Irradiation of cancerous tissue either directly destroys the cancer cells or damages them such that they cannot reproduce. One side-effect of radiotherapy is that tumor-surrounding normal tissue is inevitably also irradiated, albeit at a lower dose. The resulting long-term damage can significantly affect cognitive performance and quality of life. Many studies investigated the effect of irradiation on normal-appearing brain tissues and some of these correlated imaging findings with functional outcome. This article provides an overview of the examination of radiation-induced injuries using conventional and enhanced MRI methods and summarizes conclusions about the underlying tissue changes. Radiation-induced morphologic, microstructural, vascular, and metabolic tissue changes have been observed, in which the effect of irradiation was evident in terms of decreased perfusion and neuronal health as well as increased diffusion and atrophy.

**Abstract:**

Radiotherapy is part of the standard treatment of most primary brain tumors. Large clinical target volumes and physical characteristics of photon beams inevitably lead to irradiation of surrounding normal brain tissue. This can cause radiation-induced brain injury. In particular, late brain injury, such as cognitive dysfunction, is often irreversible and progressive over time, resulting in a significant reduction in quality of life. Since 50% of patients have survival times greater than six months, radiation-induced side effects become more relevant and need to be balanced against radiation treatment given with curative intent. To develop adequate treatment and prevention strategies, the underlying cause of radiation-induced side-effects needs to be understood. This paper provides an overview of radiation-induced changes observed in normal-appearing brains measured with conventional and advanced MRI techniques and summarizes the current findings and conclusions. Brain atrophy was observed with anatomical MRI. Changes in tissue microstructure were seen on diffusion imaging. Vascular changes were examined with perfusion-weighted imaging and susceptibility-weighted imaging. MR spectroscopy revealed decreasing N-acetyl aspartate, indicating decreased neuronal health or neuronal loss. Based on these findings, multicenter prospective studies incorporating advanced MR techniques as well as neurocognitive function tests should be designed in order to gain more evidence on radiation-induced sequelae.

## 1. Introduction

Radiotherapy (RT) is an indispensable part of the treatment of the majority of primary and metastatic brain tumors [1,2,3,4]. Several hundred thousand patients undergo radiotherapy to the brain every year [5], while 50% survive longer than six months [6]. In order to capture the expansion of infiltrative tumor and compensate for positioning uncertainties and physical limitations of the radiation technique, irradiation of tumor-surrounding normal brain tissue is unavoidable. Such irradiation of normal tissue can lead to radiation-induced brain injuries, which are categorized into acute, early delayed, and late delayed effects [7,8]. Acute and early delayed changes are usually transient and reversible, while late delayed effects are often irreversible and progressive [6,7,8,9,10,11,12]. Especially for patients surviving longer than six months, the long-term side effects can result in cognitive dysfunction and a lower quality of life [6,13,14,15,16]. To develop effective treatment and alongside prevention strategies counteracting these side effects, the underlying causes need to be understood [17,18]. Tissue changes following radiation treatment, such as contrast enhancement and T2 hyper- and hypo-intensities, are commonly observed in follow-up imaging. However, the discrimination between treatment-related changes and tumor progression remains one of the most challenging questions in brain tumor treatment [19,20]. Consequently, many studies have focused on normal-appearing brain tissue to detect and understand radiotherapy-induced tissue changes. To enable the specific determination and an early observation of radiation-induced alterations of normal brain tissue, advanced MRI techniques are often used [21,22,23,24]. Morphological changes can be investigated by evaluating conventional anatomical MR images, while changes on vasculature, microstructure, and metabolism can be detected with functional MRI methods such as perfusion-weighted imaging, diffusion-weighed imaging, and MR spectroscopy, respectively [25,26,27,28]. The aim of this paper is to review the current state of studying radiation-induced side-effects with advanced MRI techniques, in particular evaluating the effect of radiation to normal-appearing brain tissue, and to summarize the findings inferred from the MRI results regarding the underlying biologic changes.

## 2. Determination of Anatomical/Morphological Changes in Normal-Appearing Tissue after Radiotherapy

Quantitative volumetric MRI measurements have been used to observe radiation-induced anatomical differences. Several studies focused on volumetric changes of the whole brain (WB), white matter (WM), and grey matter (GM) [29,30,31,32,33,34,35,36,37], while other studies evaluated volume changes in specific brain structures such as the hippocampus [38,39,40,41,42,43,44], amygdala [44,45], corpus callosum [46], subregions of the cerebral cortex [47,48,49,50], and cerebellum [51,52]. Additionally, in some studies, the correlation of brain volume loss with cognitive decline [31,33,34,52], aging or patient age at radiation [30,32,33,36,37,42,45,46,50,51,52] was investigated. Throughout all the studies, the tissue response to radiation was always a volume decrease and never an increase. Table 1 provides an overview of the publication focusing on anatomical changes in normal tissue after radiation.

Petr et al. [36] examined volume changes within the normal-appearing hemisphere of 57 glioma patients with unilateral glioblastomas. Forty-one patients were treated with photons and showed a significant volume decrease of WM (−1.2%) and GM (−2.2%) three months after radiotherapy. Additionally, the authors examined volume changes after proton therapy in 16 patients, showing no significant decrease for the whole brain GM and WM volume, but a significant difference between low- and high-dose areas. The mean dose in the healthy hemisphere (0.9%/10 Gy), time after treatment (0.25%/month), and patient age correlated with volume loss, while no effect of gender, body mass index, clinical target volume, and duration of chemotherapy was seen. The observation of dose-dependency combined with the differences after photon and proton therapy led to the conclusion that the volumetric changes were radiation-induced. The radiation-induced GM decrease was much higher than the normal age-dependent GM loss of 0.26% to 0.39% per year in adults (aged 17 to 84 years) [53,54]. In a longitudinal MR study, Prust et al. [37] examined volume changes in the WB, GM, and WM in eight glioblastoma patients. WB and GM showed a progressive decrease, reaching significance after 27 and 23 weeks after radiotherapy respectively, independent of age, gender, and tumor volume. Contrary to Petr et al. [36], WM volume did not change. They suggested that the discrepancy to the results of Prust et al. [37] could be explained by inaccuracies in the GM and WM classification caused by segmenting the whole brain volume including abnormal tumor tissue [36]. Gommlich et al. [35] detected a significant WM volume decrease one and two years after photon irradiation of 84 glioma patients. No significant changes were found in GM and WB volumes. However, the authors acknowledge the limitations of their study, with inconsistent MR image data acquired at different scanners, field strengths, and resolution as well as differences in patient characteristics and treatment.

Several studies of the St Jude Children’s Research Hospital in Memphis focused on volume loss of normal-appearing brain tissue of medulloblastoma patients who received radiotherapy during childhood [29,30,31,32,33,34]. These studies reported no effect on GM volume, but a decreased WM volume after radiotherapy when comparing irradiated patients with healthy children [32] and with age-matched low-grade astrocytoma patients treated with surgery alone [29]. In 26 medulloblastoma patients, Reddick et al. [30] detected a correlation between WM loss and time after RT. Age-dependency was reported by Reddick et al. [32] since stronger radiation-induced WM atrophy was measured in children, who received radiotherapy at the age of six years, than in children irradiated at the age of 12 years. The underlying cause was hypothesized to be halted myelination in younger patients. For a cohort of 42 childhood medulloblastoma survivors, Mulhern and co-workers [33] associated decreased normal-appearing WM with worse neurocognitive performance, which was correlated with RT at a young age. Normal-appearing WM decrease was shown to correlate with decline in full-scale intelligence quotient (IQ), factual knowledge, verbal and nonverbal thinking, attention, and learning [31,34]. Limitations of the above publications [29,30,31,32,33,34] were problems with the partial volume effect due to the slice thickness and a stronger sensitivity to position and tilt variation as only a single transversal slice was evaluated.

Liu et al. [47] found cortical thinning in posterior parts of the brain of nine children (average age at diagnosis: 8.9 ± 3.3 years) with medulloblastoma after an average interval of 2.8 ± 2.4 years from diagnosis, independent of radiation dose. The children underwent surgery, radiotherapy, and chemotherapy. Comparing the areas of tissue changes of the medulloblastoma patients with the same areas in nine age-matched healthy controls showed that radiation-induced volume reduction occurred more often in regions of normal age-related thinning. Therefore, the authors conclude that areas undergoing age-dependent development during normal maturation might be more sensitive to the effect of radiotherapy. As no significant cortical thinning was detected in high-dose regions, the authors suggest that the cortical thinning does not primarily depend on the radiation dose. Similarly, Palmer et al. [46] examined a cohort of 35 children with medulloblastoma and reported significant radiation-induced atrophy of the corpus callosum in areas where the highest volume growth rate in normal-developing children is expected [55,56]. However, the corpus callosum was only evaluated in a single sagittal slice. The greatest volume decrease was measured in posterior subregions of corpus callosum, the isthmus and the splenium. Nagel et al. [38] detected posterior hippocampal volume reductions in 25 children with medulloblastoma one year after treatment, followed by a return to normal maturation after two to three years. They interpreted this result as an indication that the ability of neural stem cells to produce hippocampal neurons is not destroyed but disrupted by radiation.

**Figure 1:**
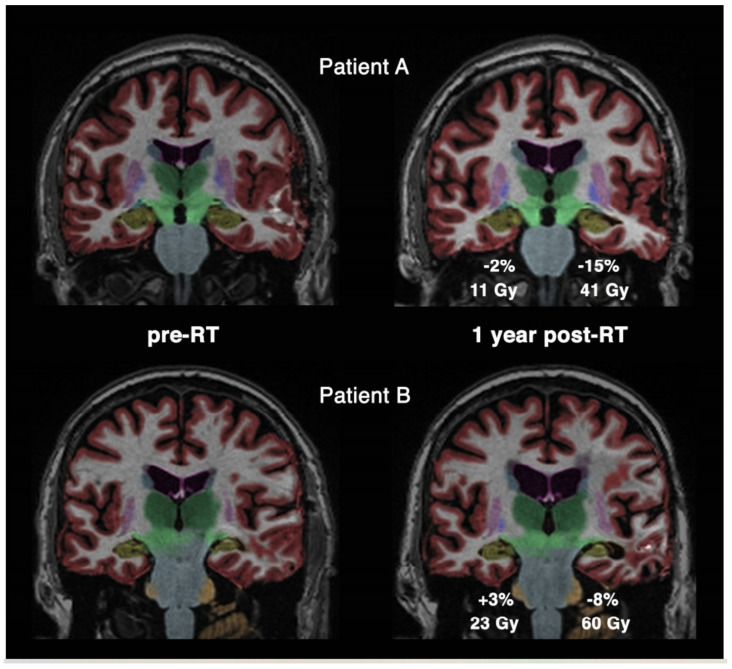
Hippocampal volume loss segmented in yellow for two cases (**A**,**B**). Comparison between measurements before radiotherapy (pre-RT) and one year after radiotherapy (post-RT) shows the different level of volume decrease for high- and low-dose irradiation. Reprinted from Seibert et al. [42] with permission from Elsevier.

Several studies focus on radiation-induced volumetric changes in the hippocampus. Takeshita et al. [41] detected hippocampal volume loss after whole brain radiation therapy with a total dose of 30 Gy in 10 fractions in 20 adult patients with lung cancer. The hippocampal volume showed a reduction of 1.8%, 5.8%, and 9.2% after 0 to 3, 4 to 7, and 8 to 11 months, respectively. Hippocampal volume loss was significantly higher than in the cortex and WM, suggesting that the hippocampus might be more vulnerable to radiation therapy. The radiation-induced hippocampal volume reduction occurred more rapidly than the volume reduction caused by Alzheimer [57]. A significant hippocampal volume loss of −6% in high-dose areas >40 Gy was reported by Seibert and colleagues [42] in 52 primary brain tumor patients one year after radiotherapy. The hippocampal decrease was age- (−0.13%/year) and dose-dependent (−0.13%/Gy). For low-dose regions <10 Gy, no significant volume change was found. Examples of the dose-dependent hippocampal volume loss in specific patient measurements can be seen in Figure 1. Similarly, no significant hippocampal volume change in low-dose areas was also reported in 15 low-dose radiated head and neck cancer patients four to ten years after RT [43]. Interestingly, Shi et al. [40] found GM atrophy only in the left hippocampus, suggesting that the left side has a larger receptor surface as the granule cells show a larger amount of dendritic segments [58]. GM was also significantly lower in the right pulvinar and the right middle temporal gyrus. In an attempt to preserve the hippocampal volume, Hong and co-workers [39] studied the effect of hippocampus-sparing RT. They reported on a volume change of +0.16% for spared hippocampi and −7.81% for non-spared hippocampi after six months.

Changes in the amygdala were focused on by Huyngh-Le et al. [45]. In a cohort of 52 patients imaged one year after RT, the authors reported on a dose-dependent volume decrease of 0.17%/Gy. Atrophy of the amygdala was associated with deviant motor behavior, potentially related to anxiety and irritability in Alzheimer patients [59]. They reported aging not to be a significant predictor for amygdala volume change, which agrees with results of MR studies investigating tissue changes in normal aging [54,60]. Still, the result is contrary to previous studies reporting age-dependent tissue changes after RT [32,33,36,42,51,52]. Nagtegaal et al. [44] determined a significant radiation dose-dependent volume loss of 0.16%/Gy to 1.37%/Gy in several GM structures, such as the amygdala, nucleus accumbens, hippocampus, globis pallidus, putamen, and thalamus, evaluating 31 patients with grade II–IV gliomas one year after RT. The only evaluated region showing no radiation effect was the caudate nucleus. The radiation-induced hippocampal volume loss was compared in a nomogram of age-dependent hippocampal volume, resulting in a mean increase in hippocampal age of 11 years in all patients.

Another study evaluated the effect of region-specific sparing in the cerebral cortex [49]. The authors found a threshold of 28.6 Gy resulting in a 20% probability of cortical atrophy one year after irradiation. Region-specific dose avoidance showed a stronger decrease of atrophy probability than non-specific cortical sparing. Seibert et al. [48] evaluated whole cortex changes in 54 patients one year after radiation and found significant dose-dependent atrophy in cortex areas connected to memory (entorhinal cortex [61]) and attention (lateral inferior parietal cortex [62]), while visual and motor regions showed no changes. The radiation-induced atrophy in areas >40 Gy was twice as high as atrophy observed in Alzheimer patients [63] and ten times the rate of normal aging [64]. Dose-dependent thinning of the temporal and limbic cortex has previously been reported [50]. These areas also showed a tendency of atrophy in Alzheimer patients [65] and therefore might be more vulnerable to injuries [50].

Two studies focused on volume changes of the cerebellum after RT [51,52]. Raschke et al. [51] found cerebellar atrophy after RT in a cohort of 91 glioma patients and a mean observational period of 428 days. Cerebellar atrophy depended significantly on time after radiotherapy, mean dose × time, and patient age. The mean dose in the cerebellum was significantly lower in patients treated with proton beam therapy compared to those treated with photons. Gender, tumor grade, and type of radiotherapy or chemotherapy were not significant predictors of cerebellar atrophy. Another group identified cerebellar atrophy changes in 25 adult survivors of cerebellar childhood tumors, presuming that young age at treatment and radiotherapy is related with cerebellar volume loss [52]. The authors found a correlation between cerebellar atrophy and oral and written processing speed. Similar results were also reported by Palmer et al. [66] in a set of 126 medulloblastoma patients aged three to 27 years, where younger age at diagnosis was significantly correlated with decreasing processing speed.

## 3. Determination of Microstructural Changes in Normal-Appearing Tissue after Radiotherapy

Diffusion imaging allows the assessment of changes in the microstructure of brain tissue in vivo. Whereas diffusion-weighted imaging (DWI) provides basic information on the apparent diffusion coefficient (ADC), also referred to as mean diffusivity (MD), diffusion-tensor imaging (DTI) provides additional information about fractional anisotropy (FA), axial diffusivity (AD), and radial diffusivity (RD). Several studies focused on microstructural tissue changes in white matter structures after radiotherapy. The most prominent effects are decreasing FA [24,67,68,69,70,71,72,73,74,75,76,77,78,79,80,81], increasing MD [24,68,70,72,77,78,79,80,81,82,83,84], and increasing RD [71,76,77,78,79,80,82,85,86,87]. AD was both measured as increasing [77,78,80,82] and decreasing [71,83,85,86,87,88,89] as a response to radiation. These diffusion changes are mostly interpreted as demyelination or axonal loss [77,80,82,83,90]. Decreasing FA and increasing MD have been interpreted as transient cerebral oedema and demyelination, whereas the inverse behavior is interpreted as recovery from oedema, oligodendrocyte regeneration, and remyelination [72]. Preclinical studies showed a correlation between RD increase and demyelination, while changes of AD indicate axonal degeneration and reactive astrogliosis [91,92]. The examined microstructural changes observed in normal-appearing tissue are summarized in Table 2.

**Figure 2:**
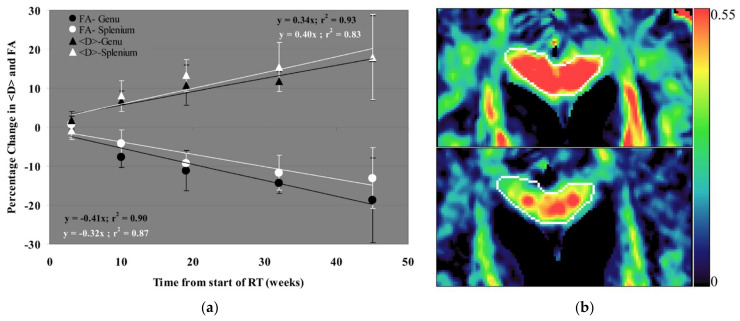
(**a**) Percentage changes of MD (here called <D>) and FA relative to pre-RT values in both genu and splenium during and after RT. (**b**) FA maps before RT (top) and 10 weeks after the start of RT (bottom) show decreasing FA in genu (outlined in white) after irradiation. Reprinted from Nagesh et al. [77] with permission from Elsevier.

Nagesh et al. [77] measured a smaller increase in AD than in RD within the normal-appearing genu and splenium in 25 patients treated with radiotherapy for low-grade, high-grade or benign tumors followed over a 45-weeks period. From their findings the authors conclude that demyelination is probably the dominant radiation-induced side-effect. The authors also recommended the examination of AD/RD ratio as a good indicator to distinguish between myelin dysfunction and axonal degradation, based on the results of preclinical studies [92,93]. Additionally, the authors reported on an increase of MD and a decrease of FA in genu and splenium. The alterations of MD and FA became significant 10 weeks after the start of RT in the genu and 19 weeks after the start of RT in the splenium. The percentage changes of MD and FA over time in both genu and splenium are shown in Figure 2a. Figure 2b displays FA maps showing the alterations in genu measured pre-RT and 10 weeks after therapy start. Xiong et al. [71] found microstructural and metabolic tissue changes in 55 nasopharyngeal carcinoma (NPC) patients within one year after radiotherapy. They published on a significant increase of RD within one year, returning to values not significantly different from pre-RT after one year. FA and AD both decreased after radiation. FA remained significantly lower than pre-RT values for all post-RT measurements and AD values were significantly decreased until nine months after treatment.

Contrary to commonly found results, the recent study by Raschke et al. [89] reported decreasing MD, RD, and AD in a cohort of 22 glioma patients and a follow-up period of between three and 24 months after the end of radio(chemo)therapy. Even though FA increased slightly, the measure for anisotropy *q* did not change. Stable values in proton density indicated that resolving oedema was not a likely cause for the decrease in diffusion. Likewise, quantitative T1 values did not change significantly; thus, changes in myelin are also an unlikely cause. A possible explanation for decreasing diffusion would be axonal swelling, similar to acute stroke [94], only at a much smaller scale. Due to reduced T2* values, Raschke et al. [89] also suggested a possible link to alterations in vascularity and tissue oxygenation based on previously reported perfusion changes in GM after radiotherapy [95]. In a follow-up study, Dünger et al. [96] could reproduce the dose- and time-dependent reduction of MD values in white matter using an independent patient cohort consisting of 70 glioma patients and clinically obtained DWI data. Decreasing RD and AD values were also observed by Zhu et al. [88] in several WM bundles of 33 patients. The degree of diffusion decrease was similar in magnitude to the values found by Raschke et al. [89] and Dünger et al. [96]. Zhu et al. [88] also found a reduction in RD and AD in nine different WM tracts following radiotherapy and, based on preclinical results [91], suggested this to be a sign for re-myelination connected with RD decrease and astrogliosis correlated with AD decrease. They examined that the change in RD and AD was significantly affected by the maximum dose to the specific WM tracts, age, and gender.

**Figure 3:**
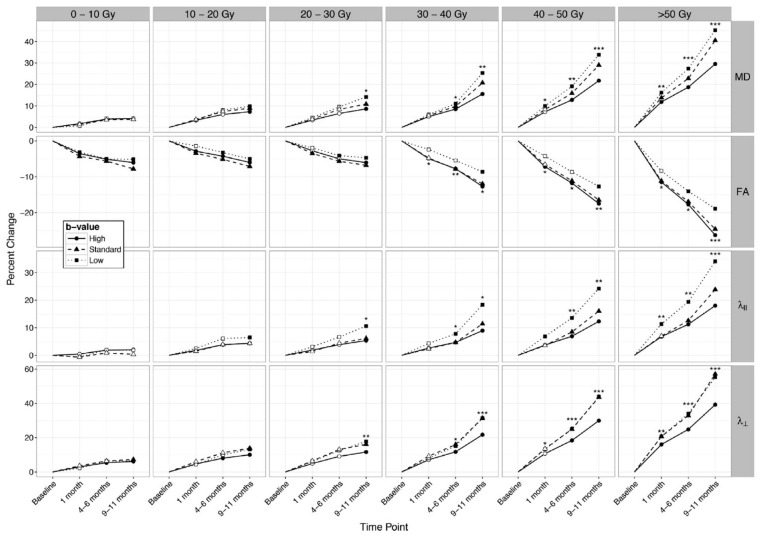
Percentage changes from baseline in MD, FA, AD (λ_‖_), and RD (λ_Ʇ_) over the time for dose-bins and different b-value measurements. All parameters show time- and dose-dependent changes. For MD, AD, and RD, the changes are most pronounced for measurements with small *b*-values, whereas for FA, the changes are most prominent at high *b*-values. Hollow points: non-significant changes, filled points: significant changes, *** *p* < 0.001, ** *p* < 0.01, * *p* < 0.05. Reprinted from Connor et al. [80] with permission from Elsevier.

Connor et al. [80] investigated the dose- and time-dependent changes of whole brain white matter in 15 glioma patients, as they measured MD, AD, and RD increase and FA decrease appearing earlier in areas receiving higher radiation doses. On average, in white matter irradiated with low doses of 10 to 20 Gy significant radiation-induced diffusion changes appeared 9 to 11 months after RT, while in areas that received > 30 Gy, the changes became significant four to six months after RT. The percentage changes of MD, FA, RD, and AD over time and in different dose regions is shown in Figure 3. Additionally, the authors measured diffusion changes using different *b*-values, which shows that MD, AD, and RD increased stronger for lower *b*-values, while the magnitude of FA decrease was greater for high *b*-values. From the stronger alterations for lower *b*-values, the authors suggested that the biological changes are prominently in the extracellular environment and are possibly caused by increased vascular permeability and neuroinflammation. Consequently, they recommended the future investigation of extracellular diffusion by measurements with different *b*-values. Dose-dependent normal-appearing WM changes were also identified by Hope et al. [82], where MD, AD, and RD were significantly increased in regions > 41 Gy compared to dose-regions < 12 Gy. In line with this, Haris et al. [83] reported that decreasing FA correlated with increasing MD only in highly radiated dose areas receiving > 50 Gy. Contrary to this, Raschke et al. [89] measured radiation-induced WM changes also in low-dose regions of 10 to 20 Gy. Nagesh et al. [77] reported a dose-dependent RD and AD increase, while Chapman et al. [86,87] did not see any correlation of tissue changes with radiation dose, gender, or age.

Connor and colleagues [79] evaluated radiation-induced changes in different white matter structures, by performing linear mixed-model analyses of the relationship between mean and maximum radiation dose, and DTI parameters. Most prominent dose-dependent changes were found in the corpus callosum, cingulum bundle, and fornix, with FA showing the strongest decrease. Several other studies reported on high radiation-induced diffusion changes in the corpus callosum, cingulum bundle, and fornix [67,76,78,82]. Two publications by the same group did not detect any changes of MD after radiotherapy of NPC patients; thus, they assumed that MD has a lower sensitivity to diffusion changes than FA [69,71]. In a small cohort of five patients, Haris et al. [83] reported an increase in MD three months after radiotherapy followed by recovery to baseline after eight months.

## 4. Determination of Vascular Changes in Normal-Appearing Tissue after Radiotherapy

### 4.1. Perfusion-Weighted Imaging

Radiation-induced vascular changes can be detected with perfusion-weighted imaging (PWI) methods such as dynamic susceptibility contrast (DSC), dynamic contrast enhancement (DCE), and arterial spin labeling (ASL). Most PWI studies found a perfusion decrease after radiotherapy, characterized by decreasing cerebral blood volume (CBV) [84,97,98,99,100], decreasing cerebral blood flow (CBF) [36,84,95,98,101], and increased K^trans^ (volume transfer constant between blood plasma and extravascular extracellular space (EES)), v_e_ (volume of EES) and v_p_ (fractional plasma volume) [102,103]. Table 3 summarizes the results of the studies focusing on vascular changes of normal-appearing brains after irradiation.

Using DSC, Wenz et al. [97] found a reduction in CBV in normal-appearing WM from 4.4 mL/100 g to 3.1 mL/100 g and in normal-appearing GM from 9.2 mL/100 g to 6.3 mL/100 g fifteen months after whole brain radiation with a dose ranging between 20 Gy and 40 Gy. In a second cohort receiving conformal radiotherapy, they reported less of a decrease in perfusion in the 30% isodose volume compared to the whole brain radiation cohort. From the same group, Fuss et al. [100] reported reduced CBV in 25 fibrillary astrocytoma patients for GM and WM within the 40 to 100% isodose volumes (mean dose 45 Gy) and a similar but not significant reduction in areas below the 40% isodose (<24 Gy). Twenty-four months after radiotherapy, CBV decreased up to 30% in high-dose areas, whereas for low-dose regions, the decrease was up to 23%, albeit non-significant. Similarly, Price et al. [98] reported on a 21% decrease in relative CBV (rCBV) and 16% decrease in relative CBF (rCBF) within the 60 to 90% dose region of WM three months after radiotherapy and no significant changes in areas outside the 60% (32 Gy) dose region. Dose-dependency appeared earlier for rCBV decrease than for rCBF decrease. The mean transit time (MTT) did not change in normal-appearing tissue. In a cohort of 17 glioblastoma patients, Fahlström et al. [101] reported a regional and global rCBV and rCBF decrease in both WM and GM until, on average, 34.4 days after RT, followed by a tendency to recover at an average of 103.3 days after RT. A correlation of the perfusion changes with dose was only detected in WM.

Contralateral GM perfusion changes using ASL were assessed by Petr et al. [95] in a cohort of 24 glioma patients with unilateral gliomas imaged three months after radio(chemo)therapy. CBF decreased in normal-appearing GM with a more pronounced decrease of 16.8% in high-dose areas > 50 Gy and a weak decrease of 2.3% in low dose volumes < 10 Gy, even so suggesting damages to the capillary bed or supplying artery. The hypothesis of radiation affecting tissue even in low-dose regions is supported by the results of Nilsen and co-authors [84], who measured decreasing vascular function with DSC after stereotactic radiosurgery (SRS) of brain metastases from non-small cell lung cancer and malignant melanoma in 40 patients. They reported decreasing CBV, CBF, and vessel density even in low-dose areas < 10 Gy at three to nine months post-SRS. In high-dose areas, the response showed similar behavior with larger variations between the patients. After 18 months, the perfusion parameter returned to pre-SRS level. Lee et al. [99] analyzed the recirculation in the time course of DSC data of 22 glioma patients before and after radiotherapy. The recirculation parameter showed a significant increase in regions above 15 Gy two months after RT. Changes of the first passage of the bolus were connected with a reduction in vascular density, while the changes in recirculation phase may indicate vessel permeability or heterogeneity increase and tortuosity [99].

As opposed to the results of most studies, Jakubovic et al. [104] reported on a significant rCBV increase in 19 metastases patients one month after SRS with the largest increase in volumes receiving 5 to 10 Gy. Relative CBF increased significantly in GM regions receiving > 10 Gy and in WM areas having undergone 5 to 16 Gy. The authors explained this early perfusion increase by early vessel dilation followed by capillary collapse or occlusion due to endothelial cell death. A perfusion increase in high-dose regions during RT has also been measured by Price et al. [98], which turned into a decrease at the end of RT.

Using DCE, Fahlström et al. [102] analyzed K^trans^ and v_e_ in WM and GM after irradiation in 17 glioblastoma patients over a period of up to 185.7 days. While global K^trans^ in WM and GM did not change significantly, v_e_ showed a significant increase in GM 101.6 days after RT. For both parameters, no dependency on the radiation dose was found. The authors interpreted the increase in v_e_ as an indicator for decreasing cell density after RT. Cao et al. [103] calculated K^trans^ and v_p_ to be significantly increased during and after RT in the cerebral tissue of 10 patients with low-grade gliomas, meningioma, cranopharyngeoma, and benign tumors. v_p_ showed a significant increase of 11% at week six during RT in high-dose areas > 40 Gy and a decrease after RT. In intermediate dose volumes of 20 to 40 Gy, the maximal increase was measured one month after RT, also followed by a decrease six months after RT. The authors interpreted the initial increase in v_p_ with fast vessel dilation during radiation [90,105,106] and the following decrease with vascular regression, such as capillary breakdown and occlusion due to endothelial cell loss, thrombus generation, or vessel renormalization [90,105,107]. K^trans^ increased significantly by 39% in medium-dose regions and 52% in high-dose regions in week six during RT. In both dose regions, K^trans^ recovered to pre-RT values six months after RT. Both parameter alterations correlated with the dose at the times of scans until one month after irradiation.

Petr et al. [36] compared the perfusion in normal-appearing GM between proton therapy and photon therapy in 19 and 47 glioblastoma patients, respectively. Three months after radio(chemo)therapy, a significant CBF decrease of −10.1% was measured in the normal-appearing hemisphere of photon-therapy patients. For proton therapy patients, the decrease of CBF was of comparable magnitude but did not reach statistical significance. Additionally, the authors could not find any correlation between perfusion decrease and radiation modality or dose.

### 4.2. Susceptibility-Weighted Imaging

**Figure 4:**
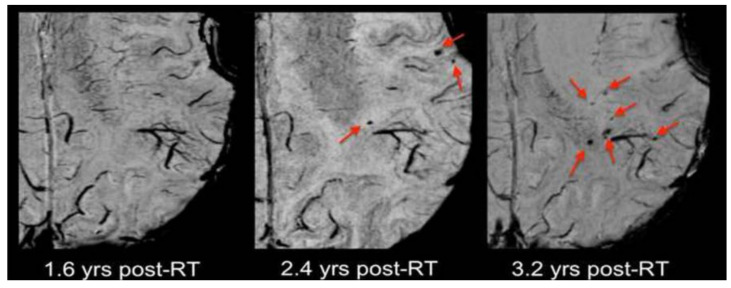
Appearance of microbleeds after RT. Microbleeds did not appear until two years after RT and increased in number (quadruple) three years after radiotherapy. Reprinted from Lupo et al. [108] with permission from Elsevier.

Few studies have assessed radiation-induced microvascular changes by visualizing microbleeds with susceptibility-weighted imaging (SWI). In a longitudinal study over 20 years, Lupo et al. [108] detected microbleeds appearing as a response to radiation in 19 glioma patients. Large numbers of microbleeds were detected. They started to appear two years after RT in high-dose regions with the majority lying within the 90% isodose region where T2-hyperintensities were detected. The number of microbleeds increased over time. After three years, microbleeds also appeared outside the T2-hyperintese areas within normal-appearing tissue. As can be taken from Figure 4, the appearance of microbleeding correlated to the time after therapy. In a second study, Lupo et al. [109] tested the effect of anti-angiogenic therapy on the appearance of radiation-induced microbleeds by observing a cohort of 17 patient with high-graded glioma between eight months to 4.5 years after RT. Results revealed a significant reduction of the initial microbleed appearance due to anti-angiogenic therapy slowing down the process. In this study, the initial onset occurred between eight and 22 months and showed a time-dependent four-fold increase after two years. Once formed, the microbleeds would not disappear again. The authors suggest that the characterization of the lesion formation in normal brain will be important to determine the vulnerability to radiation dose of different brain parts. By observing seven children between four and 62 months after radiotherapy, Peters et al. [110] showed an even faster and earlier appearance of dot-like SWI lesions in children compared to adults. The lesion appearing earliest was detected four months after RT. The authors found no correlation between the total number of SWI lesions or an increase in the number of SWI lesions over time with the radiation dose on the one hand or the patient age at the time of treatment on the other hand. Furthermore, they were unable to detect a distinct distribution of either the number of lesions or their location.

## 5. Determination of Metabolic Changes in Normal-Appearing Tissue after Radiotherapy

Magnetic resonance spectroscopy (MRS) allows for non-invasive detection of metabolic changes in the brain, and thus holds potential to quantify radiation-induced neurotoxicity. The metabolites N-acetyl aspartate (NAA), a marker for neuronal health and density; choline (Cho), a marker of membrane integrity and proliferation; and creatine (Cr), a bioenergetic metabolite [111,112], have mainly been evaluated. Some studies also investigated changes in lactate (Lac), as a marker for anaerobic metabolism [111,112], and lipids (Lip), associated with cell membrane disintegration [113,114,115]. Both the absolute metabolite values [116,117,118,119,120,121,122] and ratios between the metabolites [68,69,71,117,119,121,122,123,124,125,126,127,128] were considered. NAA/Cho mainly decreased in normal tissue after radiation [69,71,117,121,122,123,124], with a few exceptions of NAA/Cho increase [126,127] and no radiation-induced changes [128]. NAA/Cr showed a decrease [68,69,71,81,117,122,124,127,128] or no changes [121,125,126]. The behavior for the Cho/Cr ratio was less consistent, as some studies detected an increase [68,121,122,123], a decrease [126,127], or no radio-induced changes after RT [69,71,125,128]. An overview of the examined publication on metabolic changes following radiotherapy is supplied in Table 4.

Kaminaga et al. [116] reported NAA decrease in the early delayed phase in 20 patients with multiple brain metastases cancer 3.6 months after RT. Even if the underlying biological changes for the NAA decrease is unclear, the authors suggest that the decrease shows the toxic effect of radiation on neurons, as most NAA is thought to be localized in neurons and axons [129,130]. NAA decrease has previously been measured correlated to carotid stenosis, cerebellitis, epilepsy, and brain injuries [131,132,133,134]. Additionally, Cho increase was detected, which could be explained by a treatment-induced damage of oligodendrocytes resulting in the breakdown or increased turnover of myelin and cell membrane [90] containing Cho-rich phospholipids [135]. A relation between degenerated myelin sheets and oligodendrocyte injury was also suggested in an animal study after detecting demyelination in irradiated areas in histopathology [136].

Early radiation-induced metabolic changes were observed by Movsas et al. [120] three to four weeks after the end of irradiation in eight lung cancer patients who underwent whole brain irradiation. The whole brain NAA decline in patients was 10% compared to a 3% fluctuation in healthy controls. A difference in NAA decrease between prophylactic (radiation dose 30 Gy) and therapeutic (radiation dose 35 to 37.5 Gy) therapy was not detected. Chawla et al. [68] evaluated four patients with brain metastases and three patients with lung cancer and determined early metabolic changes in highly radiosensitive brain structures one month after RT. They reported significantly increasing Cho/Cr and a trend toward decreasing NAA/Cr in the hippocampus, as well as increasing Cho/Cr values in the genu of corpus callosum.

**Figure 5:**
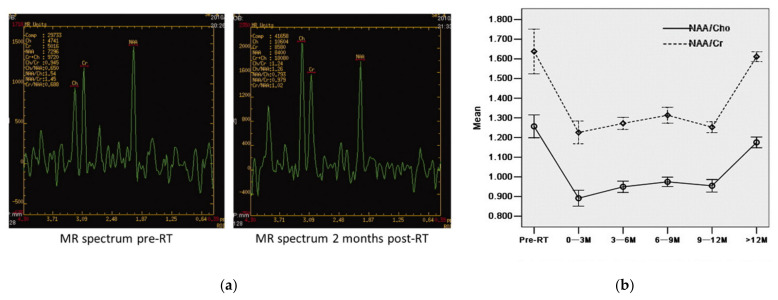
(**a**) Metabolic changes before and two months after radiotherapy. The MRS evaluation results in a decrease of NAA/Cho and NAA/Cr two months post-RT. (**b**) The curves show the alterations of NAA/Cr and NAA/Cho over time for the whole cohort. Reprinted from Xiong et al. [71] with permission from Elsevier.

In several studies, a recovery of the metabolic alteration was reported. Esteve and colleagues [117] investigated metabolic changes in the contralateral normal brain hemisphere in a cohort of 11 glioma patients (grade II–IV). They detected NAA/Cr, NAA/Cho and NAA decrease and Cho increase during the first four months after RT. Eight months after RT, the metabolic changes recovered. As NAA was involved in all alterations, the authors suggested NAA decrease as the main effect of radiotherapy. Additionally, the transient NAA/Cho and NAA/Cr decrease may indicate temporary neuronal disfunctions [117]. Similar metabolic changes have been found in a comparison of 48 NPC patients with 24 healthy controls [69]. The authors measured decreased NAA/Cr and NAA/Cho and constant Cho/Cr. NAA/Cho showed the strongest decrease in the early delayed phase (<six months after RT), which was consistent with other studies for NPC patients [137,138]. NAA/Cho and NAA/Cr partially recovered after six months, not reaching the ratios of the control cohort 12 months after RT. A possible explanation for the constant Cho/Cr ratio given by the authors could be a simultaneous Cho and Cr increase, as proliferation of glial cell causing Cho increase, but repairing of the injured cells accelerates the metabolism, leading to a Cr increase [69]. In agreement with this, Xiong et al. [71] also evaluated 55 NPC patients and detected decreasing NAA/Cho and NAA/Cr ratios during a one-year observation in the bilateral temporal lobe WM. The greatest decrease was seen three months after RT and followed by an increase 12 months after RT, albeit not fully recovering to normal values. The time-dependent alterations of NAA/Cr and NAA/Cho are presented in Figure 5b. Figure 5a also shows the spectrum displaying the metabolic changes measured before and two months after radiotherapy. The authors suggest that the NAA decrease could be caused by a shutdown of NAA synthesis [139]. As NAA did not completely recover and as neurons cannot regenerate [140], the authors proposed that a small number of neurons probably underwent cell death.

Dose-dependent metabolic changes and recovery in the first six months following radiation were detected by Lee et al. [122] in normal-appearing WM of 10 glioblastoma patients. In this study, NAA/Cho decreased significantly in low- and medium-dose areas (0 to 25 Gy, 25 to 50 Gy) two months after RT and in high-dose areas (>50 Gy) until four months after RT, followed by a dose-dependent recovery. Cho/Cr increased two months after therapy in all dose regions and declined below pre-RT values for low and medium doses. NAA/Cr fell monotonical and dose-dependent in medium and high-dose areas and showed an increase after six months in the high-dose region. Cho increase and NAA decrease was stronger in high than in low-dose regions, while Cr showed no dose-dependency. Pronounced metabolic recovery was observed by Matulewicz et al. [123] measuring oscillating metabolic changes over two years in the normal-appearing white matter of 100 patients. Three to four months after, RT NAA/Cho reached the first minimum and Cho/Cr the first maximum, followed by recovery. This process repeated every eight months. After 20 months, the ratios were close to pre-RT values. The authors proposed an early BBB disruption due to radiation followed by its repair as a possible explanation [141], which indicate that MRS could provide information about BBB permeability.

Contrary to the previous results, Sundgren et al. [127] measured decreasing Cho/Cr values in 11 patients with either low-grade glioma or benign tumors. The Cho/Cr as well as a NAA/Cr decrease already appeared in the third week of RT, while a NAA/Cho increase was detected one month after RT followed by a trend of recovering six months after RT. The metabolic changes did not show dose-dependency, but Cho/Cr correlated with the product of the dose volume receiving a dose >40 Gy and the received radiation dose. This led to the suggestion that the effect of radiation increases with the size of the volume receiving high doses.

A dose-dependent NAA/Cr decrease was reported by Rutkowski et al. [128] in 43 glioma patients nine months after RT. The NAA/Cr decrease appeared in all dose regions and was stronger for patients with total resection compared to partial or subtotal resection. They also reported Lac and Lip signal changes after RT. However, the authors used a conventional short echo-time PRESS sequence at 2T, which is highly unlikely to separate Lac and Lip signals at 1.3 ppm [142,143].

Proton (*n* = 10) and phosphorus (*n* = 13) MRS were evaluated in a cohort of 23 patients, mainly malignant gliomas, by Szigety and co-workers [121]. The sub-cohort of 10 patients showed an increase in Cho/Cr and a decrease in NAA/Cho two and four months after RT on the ipsilateral hemisphere in high-dose areas receiving 65% of the total radiation dose. The authors postulated a radiation-induced increase of Cho, but stable NAA and Cr levels. The evaluation with phosphorus MRS did not show any metabolic changes beyond the normal variation seen in healthy controls.

In addition to studies of acute and early delayed effects, other studies considered the long-term damage of radiation [118,119,125,126]. Virta et al. [126] compared late delayed metabolic changes in nine glioma patients 0.5 to 10.5 years after radiation to nine healthy controls of the same age. In normal-appearing WM, Cho/Cr was 24% lower and NAA/Cho 20% higher than in the patient cohort, while there was no difference for NAA/Cr. Metabolic differences of normal-appearing WM and hyperintense areas led to the suggestion of a 20 to 24% decrease for Cho and stable or similarly less affected values for NAA and Cr in the normal-appearing brain regions. A possible explanation given by the authors was radio-induced membrane damage in normal-appearing WM without axonal or neuronal injuries, which could result from low-dose radiation damaging myelin or myelin-producing oligodendrocytes [144]. Hypoperfusion and hypoxia resulting from endothelial radiation injury and capillary occlusion was named by the authors as another explanation, as the effect of hypoxia combined with impaired perfusion is stronger on myelin membranes than neurons [145]. Usenius et al. [119] also investigated the long-term effects in eight brain tumor patients 0.5 to 13 years after radiation. Contrary to Virta et al. [126], they only found metabolic changes in abnormal areas containing T2 hyperintensities that received a high dose, but not in medium-radiated normal-appearing brain tissue. Similar results were found by Chong et al. [118] 0.8 to 7.3 years after RT in a sub-cohort of four patients with normal imaging findings, wherein only NAA was significantly lower than in healthy controls.

Waldrop et al. [124] focused on the influence of radiotherapy and combined radiochemotherapy on the normal tissue of 70 children (mean age 10.9 years) with primary brain tumors compared to a healthy control cohort (mean age 10.4 years). They found significantly lower NAA/Cho and NAA/Cr values in grey and white matter voxel located in the right or left frontal lobe on the contralateral hemisphere in patients. A stronger NAA/Cr decrease was reported for children receiving chemotherapy as a part of the treatment, compared to no chemotherapy. Additionally, they found a stronger NAA/Cr and NAA/Cho decrease if chemotherapy was carried out before radiotherapy. Metabolic alterations were not affected by surgery but differed depending on the tumor type.

## 6. Conclusions and Summary

We summarized the current status of advanced MR imaging techniques for the assessment of the radiation-induced anatomical, morphological, and metabolic damage of normal-appearing brain tissue in primary brain tumor patients. Advanced MRI techniques offer the opportunity to visualize tissue changes not detectable with common T1- and T2-weighted MRI and highlight early tissue alterations. Anatomical and morphological damage was indicated by atrophy measured in both grey and white matter as well as in several substructures. Various studies reported on volume decrease after irradiation, which was more pronounced than that observed in normal aging or in patients with Alzheimer’s disease. Additionally, natural maturation of the brain in pediatric patients was hampered following irradiation. The effect of irradiation on the tissue microstructure was assessed using diffusion-weighted and diffusion-tensor imaging and revealed changes indicative of demyelination, axonal loss, and transient cerebral oedema. Perfusion-weighted imaging showed decreased cerebral blood flow and cerebral blood volume. Finally, metabolic changes assessed by MR spectroscopy indicated adverse effects on neuronal health, the cell membrane, and myelin.

Based on the reviewed results, future studies of normal tissue examination after irradiation may incorporate a few improvements. First, when assessing radiation damage to the brain, increased attention should be paid to the impact on the patient’s neurocognitive functioning and quality of life by implementing adequate tests and correlating results with imaging findings. Second, future studies may benefit from evaluating multiple MR contrasts in the same patient cohort, since the combination of diverse information allows more detailed conclusions on the underlying tissue alterations. Third, to improve future publications on irradiation-induced MR changes and their inter-comparability, we strongly suggest a more comprehensive description of the used methodology. In particular, it is necessary to clearly describe the exclusion criteria for abnormal tissue, especially whether and how abnormal tissue was censored at various timepoints. Fourth, multicenter studies including larger numbers of patients and obtaining data on different scanners will increase the clinical relevance of the received results. Finally, to avoid the influence of infiltrating tumor cells on the surrounding normal-appearing brain, cohorts of patients with benign brain tumors or preferably head–neck cancers may be assessed, since tissue changes of the brain can be assumed to solely stem from irradiation.

In the era of highly conformal photon beam techniques, as well as the worldwide increasing availability of proton and particle beams, advanced MRI may serve as an objective measure for the selection of brain tumor patients. This review shows the potential of MRI for normal tissue response assessment, and thus, it should be included in future prospective clinical trials.

## 7. Review Criteria

The literature search was based primarily on PUBMED combined with the consideration of secondary literature of the studied publications and google inquiries. The development of the literature collection was completed by December 2020. The data is based on research papers, while review articles were studied and consulted for further publications. Special attention by collecting publications was paid to the exclusion of abnormal tissue and the focus on normal-appearing tissue. The following shows a partial selection of the keywords used: Radiation-induced changes/injuries/alterations in brain/CNS; early and late/longitudinal radiation effects; response to radiotherapy/irradiation; cognitive impairment; normal-appearing brain tissue; conventional MRI; functional/quantitative MRI; microstructural/vascular/anatomical/morphological/metabolically changes in normal brain; Whole brain/white matter/grey matter volume change/loss/decrease; atrophy; diffusion tensor/weighted imaging; microstructure; diffusivity; demyelination; WM damage; perfusion-weighted imaging; susceptibility-weighted imaging; dynamic susceptibility contrast; dynamic contrast enhancement; arterial spin labeling; relative/absolute blood flow/volume; intra and extracellular space transition; histopathological changes; vessel dilatation; permeability; microbleeds; SWI lesion; MR spectroscopy; metabolism; NAA, neuronal marker; choline, cell membrane synthesis; creatine, energy metabolism; and several more. MR technique-specific keywords were combined with tissue alterations due to irradiation damage and the requirement for normal tissue observation with AND/OR research functions on PUBMED. Abnormal tissue changes due to irradiation were partially excluded.

## Figures and Tables

**Table 1:** Publications on anatomical/morphological changes in normal-appearing tissue after radiotherapy.

Reference	Number of Patients	Patient Age [yr]	Disease (WHO Grade)	Radiation Dose	Timing of Radiological Follow-Up	Magnetic Field Strength [T]	MRI Sequence(s)	Tissue Assessed	Main Findings/Alterations
Nagtegaal et al., 2021 [44]	31	50 ± 15	G (II–IV)	50.4–60 Gy	≥one follow-up 270–360 d post-RT	3	T1w	GM structures	Atrophy in all GM structures except caudate nucleus
Raschke et al., 2020 [51]	91	52.3 ± 14.5	G (I–IV)	54 Gy or 60 Gy	3, 6, 9, 12, 15, 18, 21 mo post-RT	3	T1w	Cerebellum	Cerebellar atrophy
Takeshita et al., 2020 [41]	20	66.2 ± 9.7	MB	30 Gy/10 fractions	0–3 mo, 4–7 mo, 8–11 mo post-RT	3	T1w	Hippocampus	Hippocampal atrophy
Huynh-Le et al., 2019 [45]	52	19–77	G (III–IV)	50.4 Gy to 60 Gy	1 y post-RT	3	T1w	Amygdala	Amygdala atrophy
Gommlich et al., 2018 [35]	26	24–74	G (II, III)	>54 Gy	no uniform time intervals	1.5 and 3	T1w	WM, GM	WM atrophy, unchanged GM volume
Petr et al., 2018 [36]	57	54.3 ± 14.2	GBM	60 Gy	3 mo and 6 mo post-RT	3	T1w	WM, GM	GM and WM atrophy
Shi et al., 2018 [40]	40	49.3 ± 11.6	NPC	12 Gy (WB) max. 72 Gy (partially)	≥12 mo post-RT	3	T1w	GM	Cortical GM atrophy in left hippocampus, right pulvinar, and right middle temporal gyrus
Seibert et al., 2017 [42]	52	19–77	primary BT	54–60 Gy	9–15 mo post-RT	3	T1w	Hippocampus	Hippocampal atrophy
Seibert et al., 2017 [48]	54	19–77	BT	54–60 Gy	9–15 mo post-RT	3	T1w	Cerebral cortex	Cortical atrophy in entorhinal and interior parietal ROIs, not in primary cortex
Ailion et al., 2016 [52]	25	9 ± 5	Cerebellar T	n.a.	15 ± 5 yr post-RT	3	T1w	Cerebellum	Cerebellar atrophy
Karunamuni et al., 2016 [49]	15	40–77	G (HG)	59.4–60 Gy	1 y post-RT	3	T1w	Cortex	RT dose above 28.6 Gy results in >20% probability of cortical atrophy
Karunamuni et al., 2015 [50]	15	40–77	G (HG)	59.4–60 Gy	1 y post-RT	3	T1w	Cortex	Cortical atrophy, strongest in temporal and limbic cortex
Hong et al., 2015 [39]	20	27–83	MBM	30 Gy/10 fractions	6 mo post-RT	n.a.	n.a.	Hippocampus	Hippocampal avoidance can minimize hippocampal atrophy
Prust et al., 2015 [37]	8	35–70	GBM	60 Gy	weekly during CRT, monthly until 6 mo pre-RT	3	T1w	WB, GM, WM, anterior lateral ventricle, hippocampal volume	WB and GM atrophy, unchanged WM and hippocampal volume, anterior lateral ventricle volume increase
Olsson et al., 2012 [43]	15	31–65	HNC	1.5–9.3 Gy	4–10 yr post-RT	1.5	T1w, T2w	Hippocampus	No hippocampal atrophy compared to healthy controls
Liu et al., 2007 [47]	9	5.4–13.9	MB	54 Gy	1.0–8.2 yr post diagnosis	1.5	T1w	Cortex	Cortical thinning
Reddick et al., 2005 [32]	52	3.5–20.0	MB	35–40 Gy	0.2–7.9 yr post-RT	1.5	T1w, T2w, PD	WM	Less developed normal appearing WM volume compared to healthy controls
Nagel et al., 2004 [38]	25	4.8–13.0	MB	23.4 Gy (average risk) 36–39.6 Gy (high risk)	0.56 yr interval post-RT	1.5	radiofrequency-spoiled, fast low-angle shot, 3D sequence	Hippocampus	Right and left hippocampal atrophy
Reddick et al., 2003 [31]	40	1.7–14.8	pediatric BT	35.2 Gy (WB) 53.1–70.2 Gy (local)	2.6–15.3 yr post-RT	1.5	T1w, T2w, PD	WM	Association between WM atrophy and reduced IQ and attentional ability
Palmer et al., 2002 [46]	35	3.2–17.2	MB	23.4 Gy (average risk) 36–39.6 Gy (high risk) 55.8 Gy (posterior fossa boost)	year 1–2: 3 mo interval, year 3–8: 6 mo interval	1.5	T1w	Corpus callosum	Decline of corpus callosum areas
Mulhern et al., 2001 [33]	42	<21	MB	49–54 Gy	≥1 yr post-RT	1.5	T1w, T2w, PD	WM	WM atrophy, young age at CRT associated with worth neurocognitive performance
Reddick et al., 2000 [30]	26	3.2–16.2	MB	36 Gy (conventional) 23.4 Gy (reduced)	mean 18.7 mo post-RT min. 4 MR examination	1.5	T1w, T2w, PD	WM	WM atrophy
Mulhern et al., 1999 [34]	18	<21	MB	23.4–36 Gy CRT 49–54 Gy (posterior fossa boost)	3.8 ± 2.6 yr post-RT	1.5	T1w, T2w, PD	WM	WM atrophy after RT may partially explain changes in IQ and cognitive function
Reddick et al., 1998 [29]	15	5.6–21	MB	25–55 Gy (WB) 55–65 Gy (focal posterior fossa)	1.2–10.6 yr post-RT	1.5	T1w, T2w, PD	WM, GM	WM atrophy, unchanged GM volume

Abbreviations: Brain metastasis (BM), Brain tumor (BT), Chemoradiotherapy (CRT), Glioma (G), Glioblastoma multiforme (GBM), Grey matter (GY), high grade (HG), Head-neck cancer (HNC), Intelligence quotient (IQ), Lung cancer (LC), low grade (LG), Melanoma (M), Medulloblastoma (MB), Melanoma brain metastasis (MBM), Mean diffusivity (MD), Nasopharyngeal carcinoma (NPC), Proton density (PD), Radial diffusivity (RD), Radiotherapy (RT), T1-weighted (T1w), T2-weighted (T2w), Tumor (T), White matter (WM), Whole brain (WB). Unit: day (y), month (mo), year (yr), Gray (Gy), Tesla (T).

**Table 2:** Publications on microstructural changes in normal-appearing tissue after radiotherapy.

Reference	Number of Patients	Patient Age [yr]	Disease (WHO Grade)	Radiation Dose	Timing of Radiological Follow-Up	Magnetic Field Strength [T]	MRI Sequence(s)	Tissue Assessed	Main Findings/Alterations
Dünger et al., 2019 [96]	70	23–82	GBM	≤60 Gy	3 mo intervals in 3–33 mo post-RT	3	DWI	WM	MD ↓
Raschke et al., 2019 [89]	22	47.8 ± 13.9	G	54–60 Gy	3, 6, 9,12,15, 18 mo post-RT	3	DTI, 2D FFE (T2*)	WM	MD ↓, RD ↓, AD ↓, T2* ↓
Tringale et al., 2019	22	20–75	primary BT	50.4–60 Gy	3, 6, 12 mo post-RT	3	DWI	Medial temporal lobe regions	MD ↑, FA ↓
Connor et al., 2017 [79]	49	24–84	G (LG, II–IV)	40.5–60 Gy	9–12 mo post-RT	3	DTI	WM tracts	MD ↑, RD ↑, FA ↓
Makola et al., 2017 [76]	14	10.1 ± 4.1	pediatric BT	52.75 Gy	3–12 mo post-surgery, follow-up 2 yr later	1.5 & 3	DTI	Corpus callosum	RD ↑, FA ↓
Chapman et al., 2016 [87]	27	26–71	LG/benign T	54 Gy	pre-RT, during RT, end of RT	3	DTI	Parahippocampal cingulum WM	AD ↓, RD ↑
Connor et al., 2016 [80]	15	40–84	G (HG)	40.05–60 Gy	1 mo, 4–6 mo, 9–11 mo post-RT	3	DTI	WM	MD ↑, AD ↑, RD ↑, FA ↓
Duan et al., 2016 [72]	81	19–65	NPC	66–74 Gy	<6 mo, 6–12 mo, >12 mo post-RT	3	DTI	WM	MD ↑, FA ↓
Zhu et al., 2016 [88]	33	25–72	LG/benign T	54 Gy	6 MRI until 18 mo post-RT	1.5 & 3	DTI	WM fiber bundles	AD ↓, RD ↓
Chawla et al., 2015 [68]	7	47–76	G (HG)	25–40 Gy	30.43 ± 9.02 d post-RT	3	DTI	Hippocampus, genu corpus callosum	MD ↑, FA ↓
Hope et al., 2015 [82]	18	33–66	G (HG)	60 Gy	every 2 w during RT, 2 w, 3 mo, 6 mo post-RT	3	DTI	WM	MD ↑, AD ↑, RD ↑
Chapman et al., 2013 [86]	14	40–76	metastases (primary: LC, M)	30 Gy/37.5 Gy	end of RT, 1 mo post-RT	3	DTI	WM structures	FA ↓, RD ↑
Xiong et al., 2013 [71]	55	19–71	NPC	66–75 Gy	0–3 mo, 3–6 mo, 6–9 mo, 9–12 mo, >12 mo post-RT	3	DTI	WM	AD ↓, RD ↑, FA ↓
Chapman et al., 2012 [85]	10	25–71	LG/benign T	54 Gy	week 3 & 6 during RT, 10, 30, 78 w post-RT	1.5	DTI	Parahippocampal cingulum bundle & temporal lobe WM	AD ↓, RD ↑
Nazem-Zadeh et al., 2012 [78]	12	53.5	BM	30 Gy/37.5 Gy	end of RT, 1 mo post-RT	3	DTI	Fiber tracts limbic circuit	MD ↑, FA ↓
Wang et al., 2012 [69]	48	16–74	NPC	68–75 Gy	<6 mo, 6–12 mo, >12 mo post-RT	3	DTI	temporal lobe	AD ↓, FA ↓
Haris et al., 2008 [83]	5	41.6 ± 11.8	G (II)	54 Gy	3, 8, 14 mo pre-RT	1.5	DTI	-	MD ↑, FA ↓
Nagesh et al., 2008 [77]	25	23–75	G (HG & LG), benign T	50–81 Gy	3, 10, 19, 32, 45 w post-RT	1.5	DTI	Genu & splenium corpus callosum	MD ↑, AD ↑, RD ↑, FA ↓
Welzel et al., 2008 [75]	16	45–67	small cell LC	30 Gy	end of RT, 6 w post-RT	1.5	DTI, T2w	Supra- & infratentorial WM	FA ↓
Qiu et al., 2007 [74]	22	8.1 ± 4.6	MB	50–55.8 Gy	3.9 ± 2.9 yr post-RT	1.5	DTI	Frontal & parietal lobes	FA ↓
Mabbott et al., 2006 [70]	8	7.5 ± 3.9	MB	55.4 Gy	2.50 ± 0.72 yr post-RT	1.5	DTI	WM	MD ↑, FA ↓
Kitahara et al., 2005 [81]	8	26–70	BT, L	30–60 Gy	0–2 mo, 3–5 mo, 6–9 mo, 10–12 mo post-RT	1.5	DTI	WM	MD ↑, FA ↓
Leung et al., 2004 [73]	16	8.8 ± 4.6	MB	50–55.8 Gy	3.1 ± 1.8 yr post-RT	1.5	DTI	WM	FA ↓
Khong et al., 2003 [67]	9	3–14	MB	50.4–54 Gy	1–6 yr post-RT	1.5	DTI	WM	FA ↓

Abbreviations: axial diffusivity (AD), brain metastasis (BM), brain tumor (BT), diffusion tensor imaging (DTI), diffusion weighted imaging (DWI), fractional anisotropy (FA), gradient echo (FFE), glioma (G), glioblastoma multiforme (GBM), high grade (HG), lymphoma (L), lung cancer (LC), low grade (LG), melanoma (M), medulloblastoma (MB), mean diffusivity (MD), nasopharyngeal carcinoma (NPC), radial diffusivity (RD), radiotherapy (RT), T2-weighted (T2w), effective T2 (T2*), tumor (T), white matter (WM), decrease (↓), increase (↑). Unit: day (y), month (mo), year (yr), gray (Gy), tesla (T).

**Table 3:** Publications on vascular changes in normal-appearing tissue after radiotherapy.

Reference	Number of Patients	Patient Age [yr]	Disease (WHO Grade)	Radiation Dose	Timing of Radiological Follow-Up	Magnetic Field Strength [T]	MRI Sequence	Tissue Assessed	Main Finding/Alteration
Nilsen et al., 2020 [84]	40	42–84	MM, metastases from non-small cell LC	15–25 Gy	3, 6, 9, 12, 18 mo post-SRS	3	DSC	GM, WM	Microvascular CBF ↓, microvascular CBV↓, vessel density ↓
Fahlström et al., 2018 [101]	10	55 ± 88	GBM (III, IV)	60 Gy	3.1, 34.4, 103.3 d post-RT	1.5	DSC	GM, WM	WM, GM: rCBV ↓, rCBF ↓
Fahlström et al., 2018 [102]	12	55.9 ± 10.8	G (III, IV)	60 Gy	3.3, 30.6, 101.6, 185.7 d post-RT	1.5	DCE	GM, WM	K^trans^ ↔, v_e_ ↔
Petr et al., 2018 [36]	67	54.9 ± 14.0	GBM	60 Gy	3, 6 mo post-RT	3	ASL	GM, WM	GM: CBF ↓
Lupo et al., 2016 [109]	17	25–66	G (HG)	n.a.	8 mo–4.5 yr post-RT	3	SWI	Brain	Appearance of microbleeds
Petr et al., 2016 [95]	24	54.3 ± 14.1	GBM	60 Gy	3, 6, 9 mo post-RT	3	ASL	GM	CBF ↓
Jakubovic et al., 2014 [104]	19	≥18	BM	16–24 Gy	1 w, 1 mo post-SRS	1.5	DSC	GM, WM	WM, GM: rCBF↑, rCBV ↑
Peters et al., 2013 [110]	7	13 ± 4	MB	29.5 Gy	4–62 mo post-RT	3	SWI	Brain	SWI lesions
Lupo et al., 2012 [108]	25	29–71	G (II–IV)	n.a.	until 20 yr post-RT	7	SWI	Brain	Appearance of microbleeds
Cao et al., 2009 [103]	10	25–71	G (LG), MG, CP, benign T	50.4–59.4 Gy	week 3, 6 during RT, 1, 6 mo post-RT	1.5	DCE	High-dose region brain	v_p_ ↑, K^trans^ ↑
Price et al., 2007 [98]	4	25–49	AA (LG)	54 Gy	after 1st fraction, end of RT, 1, 3 mo post-RT	3	DSC	Periventricular WM	rCBV↓, rCBF ↓
Lee et al., 2005 [99]	22	26–73	G (II, IV)	60 Gy	first 4 mo post-RT	1.5	DSC	WM	Recirculation phase ↓
Fuss et al., 2000 [100]	25	28–59	Fibrillary AA (II)	60–66 Gy	6 w post-RT, in 6 mo intervals	1.5	DSC	GM WM	GM/WM: CBV ↓
Wenz et al., 1996 [97]	13	40–78	Multiple intracerebral metastases, small-cell LC (prophylactic RT)	30–40 Gy	during and until 79 mo post-RT	1.5	DSC	GM WM	GM/WM: CBV ↓

Abbreviations: astrocytoma (AA), arterial spin labeling (ASL), brain metastasis (BM), cerebral blood flow (CBF), cerebral blood volume (CBV), cranopharyngioma (CP), dynamic contrast enhanced (DCE), dynamic susceptibility contrast (DSC), glioma (G), glioblastoma multiforme (GBM), grey matter (GM), proton (1H), high grade (HG), volume transfer constant between blood plasma and extravascular extracellular space (K^trans^), lung cancer (LC), low grade (LG), medulloblastoma (MB), meningioma (MG), malignant melanoma (MM), radiotherapy (RT), susceptibility-weighted imaging (SWI), stereotactic radiosurgery (SRS), tumor (T), volume of extravascular extracellular space (v_e_), fractional plasma volume (v_p_), white matter (WM), decrease (↓), increase (↑), no change (↔). Unit: day (y), month (mo), year (yr), gray (Gy), tesla (T).

**Table 4:** Publications on metabolic changes in normal-appearing tissue after radiotherapy.

Reference	Number of Patients	Patient Age [yr]	Disease (Grade)	Radiation Dose	Timing of Radiological Follow-Up	Magnetic Field Strength [T]	MRS Sequence	Tissue Assessed	Main Findings/Alterations
Chawla et al., 2015 [68]	7 (4 BM, 3 LC)	47–76	BM & LC	BM: WBRT 30–40 Gy LC: PCI 25 Gy/10 fractions	30.5 ± 9.2 d post-RT	3	3D EPSI	Bilateral GM and WM substructures	NAA/Cr ↓, Cho/Cr ↑
Xiong et al., 2013 [71]	55	19–71	NPC	66–75 Gy	0–3 mo, 3–6 mo, 6–9 mo, 9–12 mo, >12 mo post-RT	3	2D PRESS (LTE)	ROI in WM of bilateral temporal lobes	NAA/Cho ↓, NAA/Cr ↓
Wang et al., 2012 [69]	48	16–74	NPC	68–75 Gy	1 mo—7 yr, divided in <6 mo, 6–12 mo, >12 mo post-RT	3	2D PRESS (LTE)	Three voxel regions in bilateral temporal lobe WM	NAA/Cho ↓, NAA/Cr ↓
Blamek et al., 2010 [125]	2	P1: 17 P2: 13	P1: MB P2: central region T	P1: WBRT: 59.4 Gy (posterior fossa boost) & 30 Gy (craniospinal RT) P2: WBRT: 45 Gy	P1: 8 yr post-RT P2: 20 yr post-RT	n.a.	SV PRESS (STE)	P1: Voxels in cerebellum left and right; P2: Voxels in frontal left, occipital left and right	NAA/Cr ↔, Cho/Cr ↔
Sundgren et al., 2009 [127]	11	25–71	G (LG), benign T	50.4–59.4 Gy	3, 6 w during RT, 1, 6 mo post-RT	1.5	2D PRESS (LTE)	≥14 voxels in brain (no cerebellum or pons)	NAA/Cho ↑, NAA/Cr ↓, Cho/Cr ↓
Matulewicz et al., 2006 [123]	100	19–74	G (I–IV)	60 Gy	one follow-up during 2 yr post-RT	2	SV PRESS (STE)	Voxels in WM	NAA/Cho ↓, Cho/Cr ↑
Kaminaga et al., 2005 [116]	20	42–75	LC, BC and malignant L	40–50 Gy	8.5 ± 4.6 d & 3.6 ± 0.5 mo post-RT	1.5	SV PRESS (multi-TE)	ROI in occipital lobe cortex containing WM	NAA ↓, Cho ↑
Lee et al., 2004 [122]	10	54.7 ± 15.8	G (IV)	60.0 ± 6.9 Gy	end of RT, 2, 4, 6 mo post-RT	1.5	3D PRESS (LTE)	Voxels with >70% WM	NAA/Cho↓, NAA/Cr ↓, NAA ↓, Cho/Cr ↑, Cho ↑
Rutkowski et al., 2003 [128]	43	16–63	primary glial T	60 Gy	9–12 mo post-RT	2	SV PRESS (STE)	Voxel in low-, medium- and high-dose brain	NAA/Cr ↓
Chong et al., 2001 [118]	18	38–64	NPC	59.4–124.8 Gy	3–9.6 yr post-RT	2	SV PRESS (STE)	Voxels in temporal lobes	NAA ↓, Cho ↔, Cr ↔
Movsas et al., 2001 [120]	8	39–70	LC	30.0–37.5 Gy	20–46 d between baseline and follow-up	1.5	WB MRS	Average WB	NAA ↓
Virta et al., 2000 [126]	9	46–61	G (AA II–III, ODG III, GBM)	55.0–70.4 Gy	0.5–10.5 yr post-RT	1.5	Multi-slice spin echo (LTE)	ROI in WM	NAA/Cho ↑, NAA/Cr ↔, Cho/Cr ↓
Esteve et al., 1998 [117]	11	44 ± 11	G (II–IV), metastatic T	60 Gy (G), 30 Gy (WBRT after metastasectomy)	1, 4, 8 mo post-RT	1.5	SV PRESS (LTE)	VOI in contralateral hemisphere	NAA/Cho ↓, NAA/Cr ↓, NAA ↓, Cho ↑
Waldrop et al., 1998 [124]	70	2–22	primary brain neoplasms	40.0–67.2 Gy	n.a.	1.5	SV PRESS (LTE)	Voxels of right or left frontal lobe, containing WM and GM	NAA/Cho ↓, NAA/Cr ↓
Usenis et al., 1995 [119]	8	36–67	BT	59–62 Gy	0.5–13 yr post-RT	1.5	SV PRESS (LTE)	VOI in parietal, frontal, temporal, or cerebellar brain (high or medium dose)	NAA ↔, Cho ↔, Cr ↔
Szigety et al., 1993 [121]	13 (^31^P) 10 (^1^H)	24–55 (^31^P) 40 ± 11 (^1^H)	G (HG & LG), Pituitary adenoma, ODG	≤80 Gy	end of RT, 2, 4, 8, 12, 24 mo post-RT	1.5	SV STEAM (LTE) (^1^H) SV (^31^P)	Brain parenchyma (each ipsilateral high-dose and contralateral low-dose area)	NAA/Cho ↓, Cho/Cr ↑, Cho ↑

Abbreviations: astrocytoma (AA), breast cancer (BC), brain metastasis (BM), brain tumor (BT), chemical shift imaging (CSI), choline (Cho), creatine (Cr), echo planar spectroscopic imaging (EPSI), glioma (G), glioblastoma multiforme (GBM), grey matter (GM), proton (1H), high grade (HG), lymphoma (L), lung cancer (LC), low grade (LG), medulloblastoma (MB), magnetic resonance spectroscopy (MRS), N-acetylaspartate (NAA), nasopharyngeal carcinoma (NPC), oligodendroglioma (ODG), phosphor (31P), patient 1/2 (P1/P2), point-resolved spectroscopy (PRESS), radiotherapy (RT), stimulated echo acquisition mode (STEAM), single voxel (SV), echo time (TE), TE < 40 ms (STE), TE > 130 ms (LTE), tumor (T), whole brain (WB), whole brain radiotherapy (WBRT), white matter (WM), decrease (↓), increase (↑), no change (↔). Unit: day (y), month (mo), year (yr), gray (Gy), tesla (T).

## Data Availability

No new data were created or analyzed in this study. Data sharing is not applicable to this article.

## References

[1] Stupp R., Hegi M.E., Mason W.P., van den Bent M.J., Taphoorn M.J.B., Janzer R.C., Ludwin S.K., Allgeier A., Fisher B., Belanger K. (2009). Effects of radiotherapy with concomitant and adjuvant temozolomide versus radiotherapy alone on survival in glioblastoma in a randomised phase III study: 5-year analysis of the EORTC-NCIC trial. Lancet Oncol..

[2] Owonikoko T.K., Arbiser J., Zelnak A., Shu H.-K.G., Shim H., Robin A.M., Kalkanis S.N., Whitsett T.G., Salhia B., Tran N.L. (2014). Current approaches to the treatment of metastatic brain tumours. Nat. Rev. Clin. Oncol..

[3] McTyre E., Scott J., Chinnaiyan P. (2013). Whole brain radiotherapy for brain metastasis. Surg. Neurol. Int..

[4] Chang S.M., Parney I.F., Huang W., Anderson F.A., Asher A.L., Bernstein M., Lillehei K.O., Brem H., Berger M.S., Laws E.R. (2005). Patterns of care for adults with newly diagnosed malignant glioma. JAMA.

[5] Wu P.H., Coultrap S., Pinnix C., Davies K.D., Tailor R., Ang K.K., Browning M.D., Grosshans D.R. (2012). Radiation induces acute alterations in neuronal function. PLoS ONE.

[6] Makale M.T., McDonald C.R., Hattangadi-Gluth J.A., Kesari S. (2017). Mechanisms of radiotherapy-associated cognitive disability in patients with brain tumours. Nat. Rev. Neurol..

[7] Tofilon P.J., Fike J.R. (2000). The Radioresponse of the Central Nervous System: A Dynamic Process. Radiat. Res..

[8] Mildenberger M., Beach T.G., McGeer E.G., Ludgate C.M. (1990). An animal model of prophylactic cranial irradiation: Histologic effects at acute, early and delayed stages. Int. J. Radiat. Oncol. Biol. Phys..

[9] Béhin A., Delattre J.-Y. (2004). Complications of radiation therapy on the brain and spinal cord. Semin. Neurol..

[10] Miyawaki D., Murakami M., Demizu Y., Sasaki R., Niwa Y., Terashima K., Nishimura H., Hishikawa Y., Sugimura K. (2009). Brain injury after proton therapy or carbon ion therapy for head-and-neck cancer and skull base tumors. Int. J. Radiat. Oncol. Biol. Phys..

[11] Parvez K., Parvez A., Zadeh G. (2014). The diagnosis and treatment of pseudoprogression, radiation necrosis and brain tumor recurrence. Int. J. Mol. Sci..

[12] Sheline G.E., Wara W.M., Smith V. (1980). Therapeutic irradiation and brain injury. Int. J. Radiat. Oncol. Biol. Phys..

[13] Greene-Schloesser D., Robbins M.E., Peiffer A.M., Shaw E.G., Wheeler K.T., Chan M.D. (2012). Radiation-induced brain injury: A review. Front. Oncol..

[14] Frost M.H., Sloan J.A. (2002). Quality of life measurements: A soft outcome—Or is it?. Am. J. Manag. Care.

[15] Meyers C.A., Geara F., Wong P.-F., Morrison W.H. (2000). Neurocognitive effects of therapeutic irradiation for base of skull tumors. Int. J. Radiat. Oncol. Biol. Phys..

[16] Merchant T.E., Conklin H.M., Wu S., Lustig R.H., Xiong X. (2009). Late effects of conformal radiation therapy for pediatric patients with low-grade glioma: Prospective evaluation of cognitive, endocrine, and hearing deficits. J. Clin. Oncol..

[17] Barazzuol L., Coppes R.P., van Luijk P. (2020). Prevention and treatment of radiotherapy-induced side effects. Mol. Oncol..

[18] Armstrong C.L., Gyato K., Awadalla A.W., Lustig R., Tochner Z.A. (2004). A critical review of the clinical effects of therapeutic irradiation damage to the brain: The roots of controversy. Neuropsychol. Rev..

[19] Walker A.J., Ruzevick J., Malayeri A.A., Rigamonti D., Lim M., Redmond K.J., Kleinberg L. (2014). Postradiation imaging changes in the CNS: How can we differentiate between treatment effect and disease progression?. Future Oncol..

[20] Verma N., Cowperthwaite M.C., Burnett M.G., Markey M.K. (2013). Differentiating tumor recurrence from treatment necrosis: A review of neuro-oncologic imaging strategies. Neuro Oncol..

[21] Kłos J., van Laar P.J., Sinnige P.F., Enting R.H., Kramer M.C.A., van der Weide H.L., van Buchem M.A., Dierckx R.A.J.O., Borra R.J.H., van der Hoorn A. (2019). Quantifying effects of radiotherapy-induced microvascular injury; review of established and emerging brain MRI techniques. Radiother. Oncol..

[22] Welzel T., Tanner J.M. (2018). Nebenwirkungen nach Strahlentherapie in der Bildgebung. Radiologe.

[23] Yang J., Xu Z., Gao J., Liao C., Wang P., Liu Y., Ke T., Li Q., Han D. (2018). Evaluation of early acute radiation-induced brain injury: Hybrid multifunctional MRI-based study. Magn. Reson. Imaging.

[24] Tringale K.R., Nguyen T.T., Karunamuni R., Seibert T., Huynh-Le M.-P., Connor M., Moiseenko V., Gorman M.K., Marshall A., Tibbs M.D. (2019). Quantitative Imaging Biomarkers of Damage to Critical Memory Regions Are Associated With Post-Radiation Therapy Memory Performance in Brain Tumor Patients. Int. J. Radiat. Oncol. Biol. Phys..

[25] Bálentová S., Hnilicová P., Kalenská D., Baranovičová E., Muríň P., Bittšanský M., Hajtmanová E., Lehotský J., Adamkov M. (2019). Metabolic and histopathological changes in the brain and plasma of rats exposed to fractionated whole-brain irradiation. Brain Res..

[26] Tian Y., Shi Z., Yang S., Chen Y., Bao S. (2008). Changes in myelin basic protein and demyelination in the rat brain within 3 months of single 2-, 10-, or 30-Gy whole-brain radiation treatments. J. Neurosurg..

[27] Chan K.C., Khong P.-L., Cheung M.M., Wang S., Cai K.-X., Wu E.X. (2009). MRI of late microstructural and metabolic alterations in radiation-induced brain injuries. J. Magn. Reson. Imaging.

[28] Tanyildizi Y., Keweloh S., Neu M.A., Russo A., Wingerter A., Weyer-Elberich V., Stockinger M., Schmidberger H., Brockmann M.A., Faber J. (2019). Radiation-induced vascular changes in the intracranial irradiation field in medulloblastoma survivors: An MRI study. Radiother. Oncol..

[29] Reddick W.E., Mulhern R.K., Elkin T., Glass J.O., Merchant T.E., Langston J.W. (1998). A hybrid neural network analysis of subtle brain volume differences in children surviving brain tumors. Magn. Reson. Imaging.

[30] Reddickaij W.E., Russell J., Glass J.O., Xiong X., Mulhern R.K., Langston J.W., Merchant T.E., Kun L.E., Gajjar A. (2000). Subtle white matter volume differences in children treated for medulloblastoma with conventional or reduced dose craniospinal irradiation. Magn. Reson. Imaging.

[31] Reddick W.E., White H.A., Glass J.O., Wheeler G.C., Thompson S.J., Gajjar A., Leigh L., Mulhern R.K. (2003). Developmental model relating white matter volume to neurocognitive deficits in pediatric brain tumor survivors. Cancer.

[32] Reddick W.E., Glass J.O., Palmer S.L., Wu S., Gajjar A., Langston J.W., Kun L.E., Xiong X., Mulhern R.K. (2005). Atypical white matter volume development in children following craniospinal irradiation. Neuro Oncol..

[33] Mulhern R.K., Palmer S.L., Reddick W.E., Glass J.O., Kun L.E., Taylor J., Langston J., Gajjar A. (2001). Risks of young age for selected neurocognitive deficits in medulloblastoma are associated with white matter loss. J. Clin. Oncol..

[34] Mulhern R.K., Reddick W.E., Palmer S.L., Glass J.O., Elkin T.D., Kun L.E., Taylor J., Langston J., Gajjar A. (1999). Neurocognitive deficits in medulloblastoma survivors and white matter loss. Ann Neurol..

[35] Gommlich A., Raschke F., Wahl H., Troost E.G.C. (2018). Retrospective assessment of MRI-based volumetric changes of normal tissues in glioma patients following radio(chemo)therapy. Clin. Transl. Radiat. Oncol..

[36] Petr J., Platzek I., Hofheinz F., Mutsaerts H.J.M.M., Asllani I., van Osch M.J.P., Seidlitz A., Krukowski P., Gommlich A., Beuthien-Baumann B. (2018). Photon vs. proton radiochemotherapy: Effects on brain tissue volume and perfusion. Radiother. Oncol..

[37] Prust M.J., Jafari-Khouzani K., Kalpathy-Cramer J., Polaskova P., Batchelor T.T., Gerstner E.R., Dietrich J. (2015). Standard chemoradiation for glioblastoma results in progressive brain volume loss. Neurology.

[38] Nagel B.J., Palmer S.L., Reddick W.E., Glass J.O., Helton K.J., Wu S., Xiong X., Kun L.E., Gajjar A., Mulhern R.K. (2004). Abnormal hippocampal development in children with medulloblastoma treated with risk-adapted irradiation. Ajnr Am. J. Neuroradiol..

[39] Hong A., Hallock H., Valenzuela M., Lo S., Steel V., Paton E., Ng D., Jacobsen K.D., Reisse C.H., Fogarty G.B. (2015). Change in the Hippocampal Volume After Whole-Brain Radiation Therapy With or Without Hippocampal Avoidance Technique. Int. J. Radiat. Oncol. Biol. Phys..

[40] Shi L., Du F.-L., Sun Z.-W., Zhang L., Chen Y.-Y., Xie T.-M., Li P.-J., Huang S., Dong B.-Q., Zhang M.-M. (2018). Radiation-induced gray matter atrophy in patients with nasopharyngeal carcinoma after intensity modulated radiotherapy: A MRI magnetic resonance imaging voxel-based morphometry study. Quant. Imaging Med. Surg..

[41] Takeshita Y., Watanabe K., Kakeda S., Hamamura T., Sugimoto K., Masaki H., Ueda I., Igata N., Ohguri T., Korogi Y. (2020). Early volume reduction of the hippocampus after whole-brain radiation therapy: An automated brain structure segmentation study. Jpn. J. Radiol..

[42] Seibert T.M., Karunamuni R., Bartsch H., Kaifi S., Krishnan A.P., Dalia Y., Burkeen J., Murzin V., Moiseenko V., Kuperman J. (2017). Radiation Dose-Dependent Hippocampal Atrophy Detected With Longitudinal Volumetric Magnetic Resonance Imaging. Int. J. Radiat. Oncol. Biol. Phys..

[43] Olsson E., Eckerström C., Berg G., Borga M., Ekholm S., Johannsson G., Ribbelin S., Starck G., Wysocka A., Löfdahl E. (2012). Hippocampal volumes in patients exposed to low-dose radiation to the basal brain. A case-control study in long-term survivors from cancer in the head and neck region. Radiat. Oncol..

[44] Nagtegaal S.H.J., David S., Philippens M.E.P., Snijders T.J., Leemans A., Verhoeff J.J.C. (2021). Dose-dependent volume loss in subcortical deep grey matter structures after cranial radiotherapy. Clin. Transl. Radiat. Oncol..

[45] Huynh-Le M.-P., Karunamuni R., Moiseenko V., Farid N., McDonald C.R., Hattangadi-Gluth J.A., Seibert T.M. (2019). Dose-dependent atrophy of the amygdala after radiotherapy. Radiother. Oncol..

[46] Palmer S.L., Reddick W.E., Glass J.O., Gajjar A., Goloubeva O., Mulhern R.K. (2002). Decline in corpus callosum volume among pediatric patients with medulloblastoma: Longitudinal MR imaging study. Ajnr Am. J. Neuroradiol..

[47] Liu A.K., Marcus K.J., Fischl B., Grant P.E., Poussaint T.Y., Rivkin M.J., Davis P., Tarbell N.J., Yock T.I. (2007). Changes in cerebral cortex of children treated for medulloblastoma. Int. J. Radiat. Oncol. Biol. Phys..

[48] Seibert T.M., Karunamuni R., Kaifi S., Burkeen J., Connor M., Krishnan A.P., White N.S., Farid N., Bartsch H., Murzin V. (2017). Cerebral Cortex Regions Selectively Vulnerable to Radiation Dose-Dependent Atrophy. Int. J. Radiat. Oncol. Biol. Phys..

[49] Karunamuni R.A., Moore K.L., Seibert T.M., Li N., White N.S., Bartsch H., Carmona R., Marshall D., McDonald C.R., Farid N. (2016). Radiation sparing of cerebral cortex in brain tumor patients using quantitative neuroimaging. Radiother. Oncol..

[50] Karunamuni R., Bartsch H., White N.S., Moiseenko V., Carmona R., Marshall D.C., Seibert T.M., McDonald C.R., Farid N., Krishnan A. (2016). Dose-Dependent Cortical Thinning After Partial Brain Irradiation in High-Grade Glioma. Int. J. Radiat. Oncol. Biol. Phys..

[51] Raschke F., Seidlitz A., Wesemann T., Löck S., Jentsch C., Platzek I., Petr J., van den Hoff J., Kotzerke J., Beuthien-Baumann B. (2020). Dose dependent cerebellar atrophy in glioma patients after radio(chemo)therapy. Radiother. Oncol..

[52] Ailion A.S., King T.Z., Wang L., Fox M.E., Mao H., Morris R.M., Crosson B. (2016). Cerebellar Atrophy in Adult Survivors of Childhood Cerebellar Tumor. J. Int. Neuropsychol. Soc..

[53] Scahill R.I., Frost C., Jenkins R., Whitwell J.L., Rossor M.N., Fox N.C. (2003). A longitudinal study of brain volume changes in normal aging using serial registered magnetic resonance imaging. Arch. Neurol..

[54] Good C.D., Johnsrude I.S., Ashburner J., Henson R.N., Friston K.J., Frackowiak R.S. (2001). A voxel-based morphometric study of ageing in 465 normal adult human brains. Neuroimage.

[55] Giedd J.N., Blumenthal J., Jeffries N.O., Rajapakse J.C., Vaituzis A., Liu H., Berry Y.C., Tobin M., Nelson J., Castellanos F. (1999). Development of the human corpus callosum during childhood and adolescence: A longitudinal MRI study. Prog. Neuro Psychopharmacol. Biol. Psychiatry.

[56] Giedd J.N., Rumsey J.M., Castellanos F., Rajapakse J.C., Kaysen D., Catherine Vaituzis A., Vauss Y.C., Hamburger S.D., Rapoport J.L. (1996). A quantitative MRI study of the corpus callosum in children and adolescents. Dev. Brain Res..

[57] Schuff N., Woerner N., Boreta L., Kornfield T., Shaw L.M., Trojanowski J.Q., Thompson P.M., Jack C.R., Weiner M.W. (2009). MRI of hippocampal volume loss in early Alzheimer’s disease in relation to ApoE genotype and biomarkers. Brain.

[58] Sá M.J., Ruela C., Madeira M.D. (2007). Dendritic right/left asymmetries in the neurons of the human hippocampal formation: A quantitative Golgi study. Arq. Neuropsiquiatr..

[59] Poulin S.P., Dautoff R., Morris J.C., Barrett L.F., Dickerson B.C. (2011). Amygdala atrophy is prominent in early Alzheimer’s disease and relates to symptom severity. Psychiatry Res..

[60] Soininen H.S., Partanen K., Pitkänen A., Vainio P., Hänninen T., Hallikainen M., Koivisto K., Riekkinen P.J. (1994). Volumetric MRI analysis of the amygdala and the hippocampus in subjects with age-associated memory impairment: Correlation to visual and verbal memory. Neurology.

[61] Kandel E.R., Schwartz J.H., Jessell T.M. (2000). Principles of Neural Science.

[62] Cabeza R., Nyberg L. (2000). Imaging cognition II: An empirical review of 275 PET and fMRI studies. J. Cogn. Neurosci..

[63] Sabuncu M.R., Desikan R.S., Sepulcre J., Yeo B.T.T., Liu H., Schmansky N.J., Reuter M., Weiner M.W., Buckner R.L., Sperling R.A. (2011). The dynamics of cortical and hippocampal atrophy in Alzheimer disease. Arch. Neurol..

[64] Salat D.H., Buckner R.L., Snyder A.Z., Greve D.N., Desikan R.S.R., Busa E., Morris J.C., Dale A.M., Fischl B. (2004). Thinning of the cerebral cortex in aging. Cereb. Cortex.

[65] Brun A., Englund E. (1981). Regional pattern of degeneration in Alzheimer’s disease: Neuronal loss and histopathological grading. Histopathology.

[66] Palmer S.L., Armstrong C., Onar-Thomas A., Wu S., Wallace D., Bonner M.J., Schreiber J., Swain M., Chapieski L., Mabbott D. (2013). Processing speed, attention, and working memory after treatment for medulloblastoma: An international, prospective, and longitudinal study. J. Clin. Oncol..

[67] Khong P.-L., Kwong D.L.W., Chan G.C.F., Sham J.S.T., Chan F.-L., Ooi G.-C. (2003). Diffusion-tensor imaging for the detection and quantification of treatment-induced white matter injury in children with medulloblastoma: A pilot study. Ajnr Am. J. Neuroradiol..

[68] Chawla S., Wang S., Kim S., Sheriff S., Lee P., Rengan R., Lin A., Melhem E., Maudsley A., Poptani H. (2015). Radiation injury to the normal brain measured by 3D-echo-planar spectroscopic imaging and diffusion tensor imaging: Initial experience. J. Neuroimaging.

[69] Wang H.-Z., Qiu S.-J., Lv X.-F., Wang Y.-Y., Liang Y., Xiong W.-F., Ouyang Z.-B. (2012). Diffusion tensor imaging and 1H-MRS study on radiation-induced brain injury after nasopharyngeal carcinoma radiotherapy. Clin. Radiol..

[70] Mabbott D.J., Noseworthy M.D., Bouffet E., Rockel C., Laughlin S. (2006). Diffusion tensor imaging of white matter after cranial radiation in children for medulloblastoma: Correlation with IQ. Neuro Oncol..

[71] Xiong W.F., Qiu S.J., Wang H.Z., Lv X.F. (2013). 1H-MR spectroscopy and diffusion tensor imaging of normal-appearing temporal white matter in patients with nasopharyngeal carcinoma after irradiation: Initial experience. J. Magn. Reson. Imaging.

[72] Duan F., Cheng J., Jiang J., Chang J., Zhang Y., Qiu S. (2016). Whole-brain changes in white matter microstructure after radiotherapy for nasopharyngeal carcinoma: A diffusion tensor imaging study. Eur. Arch. Otorhinolaryngol..

[73] Leung L.H.T., Ooi G.C., Kwong D.L.W., Chan G.C.F., Cao G., Khong P.L. (2004). White-matter diffusion anisotropy after chemo-irradiation: A statistical parametric mapping study and histogram analysis. Neuroimage.

[74] Qiu D., Kwong D.L.W., Chan G.C.F., Leung L.H.T., Khong P.-L. (2007). Diffusion tensor magnetic resonance imaging finding of discrepant fractional anisotropy between the frontal and parietal lobes after whole-brain irradiation in childhood medulloblastoma survivors: Reflection of regional white matter radiosensitivity?. Int. J. Radiat. Oncol. Biol. Phys..

[75] Welzel T., Niethammer A., Mende U., Heiland S., Wenz F., Debus J., Krempien R. (2008). Diffusion tensor imaging screening of radiation-induced changes in the white matter after prophylactic cranial irradiation of patients with small cell lung cancer: First results of a prospective study. Ajnr Am. J. Neuroradiol..

[76] Makola M., Douglas Ris M., Mahone E.M., Yeates K.O., Cecil K.M. (2017). Long-term effects of radiation therapy on white matter of the corpus callosum: A diffusion tensor imaging study in children. Pediatr. Radiol..

[77] Nagesh V., Tsien C.I., Chenevert T.L., Ross B.D., Lawrence T.S., Junick L., Cao Y. (2008). Radiation-induced changes in normal-appearing white matter in patients with cerebral tumors: A diffusion tensor imaging study. Int. J. Radiat. Oncol. Biol. Phys..

[78] Nazem-Zadeh M.-R., Chapman C.H., Lawrence T.L., Tsien C.I., Cao Y. (2012). Radiation therapy effects on white matter fiber tracts of the limbic circuit. Med. Phys..

[79] Connor M., Karunamuni R., McDonald C., Seibert T., White N., Moiseenko V., Bartsch H., Farid N., Kuperman J., Krishnan A. (2017). Regional susceptibility to dose-dependent white matter damage after brain radiotherapy. Radiother. Oncol..

[80] Connor M., Karunamuni R., McDonald C., White N., Pettersson N., Moiseenko V., Seibert T., Marshall D., Cervino L., Bartsch H. (2016). Dose-dependent white matter damage after brain radiotherapy. Radiother. Oncol..

[81] Kitahara S., Nakasu S., Murata K., Sho K., Ito R. (2005). Evaluation of treatment-induced cerebral white matter injury by using diffusion-tensor MR imaging: Initial experience. Ajnr Am. J. Neuroradiol..

[82] Hope T.R., Vardal J., Bjørnerud A., Larsson C., Arnesen M.R., Salo R.A., Groote I.R. (2015). Serial diffusion tensor imaging for early detection of radiation-induced injuries to normal-appearing white matter in high-grade glioma patients. J. Magn. Reson. Imaging.

[83] Haris M., Kumar S., Raj M.K., Das K.J.M., Sapru S., Behari S., Rathore R.K.S., Narayana P.A., Gupta R.K. (2008). Serial diffusion tensor imaging to characterize radiation-induced changes in normal-appearing white matter following radiotherapy in patients with adult low-grade gliomas. Radiat. Med..

[84] Nilsen L.B., Digernes I., Grøvik E., Saxhaug C., Latysheva A., Geier O., Breivik B., Sætre D.O., Jacobsen K.D., Helland Å. (2020). Responses in the diffusivity and vascular function of the irradiated normal brain are seen up until 18 months following SRS of brain metastases. Neurooncol. Adv..

[85] Chapman C.H., Nagesh V., Sundgren P.C., Buchtel H., Chenevert T.L., Junck L., Lawrence T.S., Tsien C.I., Cao Y. (2012). Diffusion tensor imaging of normal-appearing white matter as biomarker for radiation-induced late delayed cognitive decline. Int. J. Radiat. Oncol. Biol. Phys..

[86] Chapman C.H., Nazem-Zadeh M., Lee O.E., Schipper M.J., Tsien C.I., Lawrence T.S., Cao Y. (2013). Regional variation in brain white matter diffusion index changes following chemoradiotherapy: A prospective study using tract-based spatial statistics. PLoS ONE.

[87] Chapman C.H., Zhu T., Nazem-Zadeh M., Tao Y., Buchtel H.A., Tsien C.I., Lawrence T.S., Cao Y. (2016). Diffusion tensor imaging predicts cognitive function change following partial brain radiotherapy for low-grade and benign tumors. Radiother. Oncol..

[88] Zhu T., Chapman C.H., Tsien C., Kim M., Spratt D.E., Lawrence T.S., Cao Y. (2016). Effect of the Maximum Dose on White Matter Fiber Bundles Using Longitudinal Diffusion Tensor Imaging. Int. J. Radiat. Oncol. Biol. Phys..

[89] Raschke F., Wesemann T., Wahl H., Appold S., Krause M., Linn J., Troost E.G.C. (2019). Reduced diffusion in normal appearing white matter of glioma patients following radio(chemo)therapy. Radiother. Oncol..

[90] Belka C., Budach W., Kortmann R.D., Bamberg M. (2001). Radiation induced CNS toxicity--molecular and cellular mechanisms. Br. J. Cancer.

[91] Wang S., Wu E.X., Qiu D., Leung L.H.T., Lau H.-F., Khong P.-L. (2009). Longitudinal diffusion tensor magnetic resonance imaging study of radiation-induced white matter damage in a rat model. Cancer Res..

[92] Song S.-K., Sun S.-W., Ju W.-K., Lin S.-J., Cross A.H., Neufeld A.H. (2003). Diffusion tensor imaging detects and differentiates axon and myelin degeneration in mouse optic nerve after retinal ischemia. Neuroimage.

[93] Song S.-K., Sun S.-W., Ramsbottom M.J., Chang C., Russell J., Cross A.H. (2002). Dysmyelination revealed through MRI as increased radial (but unchanged axial) diffusion of water. Neuroimage.

[94] Fung S.H., Roccatagliata L., Gonzalez R.G., Schaefer P.W. (2011). MR diffusion imaging in ischemic stroke. Neuroimaging Clin. N. Am..

[95] Petr J., Platzek I., Seidlitz A., Mutsaerts H.J.M.M., Hofheinz F., Schramm G., Maus J., Beuthien-Baumann B., Krause M., van den Hoff J. (2016). Early and late effects of radiochemotherapy on cerebral blood flow in glioblastoma patients measured with non-invasive perfusion MRI. Radiother. Oncol..

[96] Dünger L. (2019). Analyse dosisabhängiger Veränderungen der Magnetresonanz-Diffusion in der weißen Substanz bei Patienten mit hirneigenen Tumoren nach erfolgter Photonen- oder Protonentherapie. M. Sc. Thesis.

[97] Wenz F., Rempp K., Hess T., Debus J., Brix G., Engenhart R., Knopp M.V., van Kaick G., Wannenmacher M. (1996). Effect of radiation on blood volume in low-grade astrocytomas and normal brain tissue: Quantification with dynamic susceptibility contrast MR imaging. Ajr Am. J. Roentgenol..

[98] Price S.J., Jena R., Green H.A.L., Kirkby N.F., Lynch A.G., Coles C.E., Pickard J.D., Gillard J.H., Burnet N.G. (2007). Early radiotherapy dose response and lack of hypersensitivity effect in normal brain tissue: A sequential dynamic susceptibility imaging study of cerebral perfusion. Clin. Oncol. (R Coll. Radiol.).

[99] Lee M.C., Cha S., Chang S.M., Nelson S.J. (2005). Dynamic susceptibility contrast perfusion imaging of radiation effects in normal-appearing brain tissue: Changes in the first-pass and recirculation phases. J. Magn. Reson. Imaging.

[100] Fuss M., Wenz F., Scholdei R., Essig M., Debus J., Knopp M.V., Wannenmacher M. (2000). Radiation-induced regional cerebral blood volume (rCBV) changes in normal brain and low-grade astrocytomas: Quantification and time and dose-dependent occurrence. Int. J. Radiat. Oncol. Biol. Phys..

[101] Fahlström M., Blomquist E., Nyholm T., Larsson E.-M. (2018). Perfusion Magnetic Resonance Imaging Changes in Normal Appearing Brain Tissue after Radiotherapy in Glioblastoma Patients may Confound Longitudinal Evaluation of Treatment Response. Radiol. Oncol..

[102] Fahlström M., Fransson S., Blomquist E., Nyholm T., Larsson E.-M. (2018). Dynamic contrast-enhanced magnetic resonance imaging may act as a biomarker for vascular damage in normal appearing brain tissue after radiotherapy in patients with glioblastoma. ACTA Radiol. Open.

[103] Cao Y., Tsien C.I., Sundgren P.C., Nagesh V., Normolle D., Buchtel H., Junck L., Lawrence T.S. (2009). Dynamic contrast-enhanced magnetic resonance imaging as a biomarker for prediction of radiation-induced neurocognitive dysfunction. Clin. Cancer Res..

[104] Jakubovic R., Sahgal A., Ruschin M., Pejovic-Milic A., Milwid R., Aviv R.I. (2014). Non Tumor Perfusion Changes Following Stereotactic Radiosurgery to Brain Metastases. Technol. Cancer Res. Treat..

[105] Ljubimova N.V., Levitman M.K., Plotnikova E.D., Eidus L.K. (1991). Endothelial cell population dynamics in rat brain after local irradiation. Br. J. Radiol..

[106] Calvo W., Hopewell J.W., Reinhold H.S., Yeung T.K. (1988). Time- and dose-related changes in the white matter of the rat brain after single doses of X rays. Br. J. Radiol..

[107] Brown W.R., Thore C.R., Moody D.M., Robbins M.E., Wheeler K.T. (2005). Vascular damage after fractionated whole-brain irradiation in rats. Radiat. Res..

[108] Lupo J.M., Chuang C.F., Chang S.M., Barani I.J., Jimenez B., Hess C.P., Nelson S.J. (2012). 7-Tesla susceptibility-weighted imaging to assess the effects of radiotherapy on normal-appearing brain in patients with glioma. Int. J. Radiat. Oncol. Biol. Phys..

[109] Lupo J.M., Molinaro A.M., Essock-Burns E., Butowski N., Chang S.M., Cha S., Nelson S.J. (2016). The effects of anti-angiogenic therapy on the formation of radiation-induced microbleeds in normal brain tissue of patients with glioma. Neuro Oncol..

[110] Peters S., Pahl R., Claviez A., Jansen O. (2013). Detection of irreversible changes in susceptibility-weighted images after whole-brain irradiation of children. Neuroradiology.

[111] Moats R.A., Watson L., Shonk T., Tokuyama S., Braslau D., Eto R., Mandigo J.C., Ross B.D. (1995). Added value of automated clinical proton MR spectroscopy of the brain. J. Comput. Assist. Tomogr..

[112] Wang Z., Zimmerman R.A., Sauter R. (1996). Proton MR spectroscopy of the brain: Clinically useful information obtained in assessing CNS diseases in children. Ajr Am. J. Roentgenol..

[113] Negendank W.G., Sauter R., Brown T.R., Evelhoch J.L., Falini A., Gotsis E.D., Heerschap A., Kamada K., Lee B.C., Mengeot M.M. (1996). Proton magnetic resonance spectroscopy in patients with glial tumors: A multicenter study. J. Neurosurg..

[114] Kizu O., Naruse S., Furuya S., Morishita H., Ide M., Maeda T., Ueda S. (1998). Application of proton chemical shift imaging in monitoring of gamma knife radiosurgery on brain tumors. Magn. Reson. Imaging.

[115] Rand S.D., Prost R., Haughton V., Mark L., Strainer J., Johansen J., Kim T.A., Chetty V.K., Mueller W., Meyer G. (1997). Accuracy of single-voxel proton MR spectroscopy in distinguishing neoplastic from nonneoplastic brain lesions. AJNR Am. J. Neuroradiol..

[116] Kaminaga T., Shirai K. (2005). Radiation-induced brain metabolic changes in the acute and early delayed phase detected with quantitative proton magnetic resonance spectroscopy. J. Comput. Assist. Tomogr..

[117] Estève F., Rubin C., Grand S., Kolodié H., Le Bas J.-F. (1998). Transient metabolic changes observed with proton MR spectroscopy in normal human brain after radiation therapy. Int. J. Radiat. Oncol. Biol. Phys..

[118] Chong V.F., Rumpel H., Fan Y.F., Mukherji S.K. (2001). Temporal lobe changes following radiation therapy: Imaging and proton MR spectroscopic findings. Eur. Radiol..

[119] Usenius T., Usenius J.-P., Tenhunen M., Vainio P., Johansson R., Soimakallio S., Kauppinen R. (1995). Radiation-induced changes in human brain metabolites as studied by 1H nuclear magnetic resonance spectroscopy in vivo. Int. J. Radiat. Oncol. Biol. Phys..

[120] Movsas B., Li B.S., Babb J.S., Fowble B.L., Nicolaou N., Gonen O. (2001). Quantifying radiation therapy-induced brain injury with whole-brain proton MR spectroscopy: Initial observations. Radiology.

[121] Szigety S.K., Allen P.S., Huyser-Wierenga D., Urtasun R.C. (1993). The effect of radiation on normal human CNS as detected by NMR spectroscopy. Int. J. Radiat. Oncol. Biol. Phys..

[122] Lee M.C., Pirzkall A., McKnight T.R., Nelson S.J. (2004). 1H-MRSI of radiation effects in normal-appearing white matter: Dose-dependence and impact on automated spectral classification. J. Magn. Reson. Imaging.

[123] Matulewicz Ł., Sokół M., Michnik A., Wydmański J. (2006). Long-term normal-appearing brain tissue monitoring after irradiation using proton magnetic resonance spectroscopy in vivo: Statistical analysis of a large group of patients. Int. J. Radiat. Oncol. Biol. Phys..

[124] Waldrop S.M., Davis P.C., Padgett C.A., Shapiro M.B., Morris R. (1998). Treatment of brain tumors in children is associated with abnormal MR spectroscopic ratios in brain tissue remote from the tumor site. AJNR Am. J. Neuroradiol..

[125] Blamek S., Wydmański J., Sokół M., Matulewicz L., Boguszewicz L. (2010). Magnetic resonance spectroscopic evaluation of brain tissue metabolism after irradiation for pediatric brain tumors in long-term survivors: A report of two cases. Acta Neurochir. Suppl..

[126] Virta A., Patronas N., Raman R., Dwyer A., Barnett A., Bonavita S., Tedeschi G., Lundbom N. (2000). Spectroscopic imaging of radiation-induced effects in the white matter of glioma patients. Magn. Reson. Imaging.

[127] Sundgren P.C., Nagesh V., Elias A., Tsien C., Junck L., Gomez Hassan D.M., Lawrence T.S., Chenevert T.L., Rogers L., McKeever P. (2009). Metabolic alterations: A biomarker for radiation-induced normal brain injury-an MR spectroscopy study. J. Magn. Reson. Imaging.

[128] Rutkowski T., Tarnawski R., Sokol M., Maciejewski B. (2003). 1h-mr spectroscopy of normal brain tissue before and after postoperative radiotherapy because of primary brain tumors. Int. J. Radiat. Oncol. Biol. Phys..

[129] Simmons M.L., Frondoza C.G., Coyle J.T. (1991). Immunocytochemical localization of N-acetyl-aspartate with monoclonal antibodies. Neuroscience.

[130] Moffett J.R., Namboodiri M.A., Cangro C.B., Neale J.H. (1991). Immunohistochemical localization of N-acetylaspartate in rat brain. Neuroreport.

[131] Guerrini L., Belli G., Cellerini M., Nencini P., Mascalchi M. (2002). Proton MR spectroscopy of cerebellitis. Magn. Reson. Imaging.

[132] Demougeot C., Garnier P., Mossiat C., Bertrand N., Giroud M., Beley A., Marie C. (2001). N-Acetylaspartate, a marker of both cellular dysfunction and neuronal loss: Its relevance to studies of acute brain injury. J. Neurochem..

[133] Cendes F., Andermann F., Preul M.C., Arnold D.L. (1994). Lateralization of temporal lobe epilepsy based on regional metabolic abnormalities in proton magnetic resonance spectroscopic images. Ann. Neurol..

[134] Uno M., Harada M., Nagahiro S. (2001). Quantitative evaluation of cerebral metabolites and cerebral blood flow in patients with carotid stenosis. Neurol. Res..

[135] Hida K., Kwee I.L., Nakada T. (1992). In vivo 1H and 31P NMR spectroscopy of the developing rat brain. Magn. Reson. Med..

[136] Rubin P., Whitaker J.N., Ceckler T.L., Nelson D., Gregory P.K., Baggs R.B., Constine L.S., Herman P.K. (1988). Myelin basic protein and magnetic resonance imaging for diagnosing radiation myelopathy. Int. J. Radiat. Oncol. Biol. Phys..

[137] Zhang X.L., Jiang M., Qiu S.J., Zhang Y. (2004). An 1H-MRS study on radioencephalopathy caused by radiotherapy of nasopharyngeal carcinoma. Chin. J. Radiol..

[138] Song Q., Xia L.M., Wang C.Y., Feng D.-Y. (2006). 1H-MRS study on radiation induced injury of the brain in early acute reaction stage after nasopharyngeal carcinoma radiotherapy. Chin. J. Radiol..

[139] Shemesh N., Sadan O., Melamed E., Offen D., Cohen Y. (2010). Longitudinal MRI and MRSI characterization of the quinolinic acid rat model for excitotoxicity: Peculiar apparent diffusion coefficients and recovery of N-acetyl aspartate levels. Nmr Biomed..

[140] Wilding C.S., Cadwell K., Tawn E.J., Relton C.L., Taylor G.A., Chinnery P.F., Turnbull D.M. (2006). Mitochondrial DNA mutations in individuals occupationally exposed to ionizing radiation. Radiat. Res..

[141] Wong C.S., van der Kogel A.J. (2004). Mechanisms of radiation injury to the central nervous system: Implications for neuroprotection. Mol. Interv..

[142] Lange T., Dydak U., Roberts T.P.L., Rowley H.A., Bjeljac M., Boesiger P. (2006). Pitfalls in lactate measurements at 3T. AJNR Am. J. Neuroradiol..

[143] Charles-Edwards G.D., Jan W., To M., Maxwell D., Keevil S.F., Robinson R. (2010). Non-invasive detection and quantification of human foetal brain lactate in utero by magnetic resonance spectroscopy. Prenat. Diagn..

[144] Rubin P., Constine L., Nelson D.F., Perez C.A., Brady L.W. (2014). Principles and Practice in Radiation Oncology. ALERT Adverse Late Effects of Cancer Treatment.

[145] Englund E., Brun A. (1990). White matter changes in dementia of Alzheimer’s type: The difference in vulnerability between cell compartments. Histopathology.

